# Enantioselective Synthesis of the Guaipyridine Alkaloid (+)- and (−)-Cananodine

**DOI:** 10.1021/acsomega.3c07735

**Published:** 2024-02-07

**Authors:** Haley M. Holliday, Kendelyn I. Bone, Rhemrose Sabio, James R. Vyvyan

**Affiliations:** Department of Chemistry, Western Washington University, Bellingham, Washington 98225, United States

## Abstract

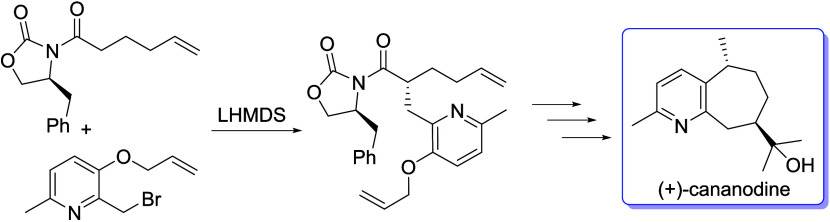

Synthesis of both enantiomers of guaipyridine alkaloid cananodine was achieved. The stereocenter at C8 was set through an Evans alkylation, and the seven-membered carbocycle was constructed using an intramolecular Mizoroki–Heck reaction. Hydrogenation of an exomethylene set the C5 stereocenter. The optical rotation of each enantiomer matched the literature. The synthetic scheme is amenable to analogue preparation. (+)- and (−)-Rupestine G were also prepared.

## Introduction

**Figure 1:**
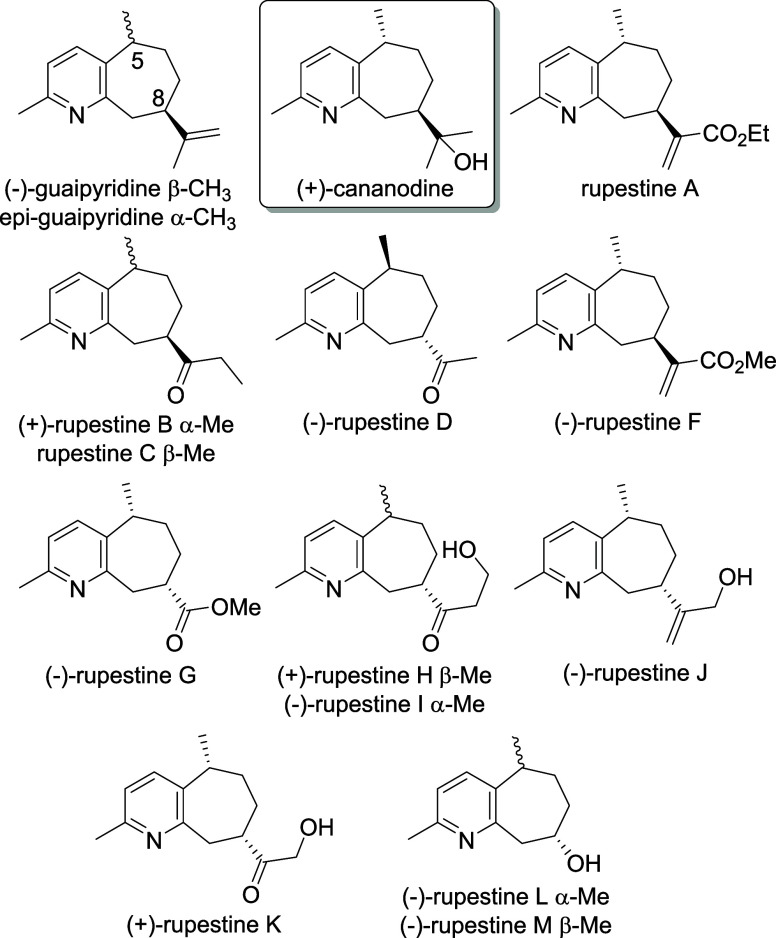
Guaipyridine alkaloids.

Cananodine (Figure 1) is a guaipyridine alkaloid isolated in small amounts from the fruit of *Cananga odorata* (ylang ylang) by Wu and co-workers, as reported in 2001.^1^ It was initially reported to have submicromolar activity against two hepatocellular carcinoma cell lines. A more recent study has shown the bioactivity to be lower than initially reported.^2^ Cananodine also has a modest activity against HeLa and MDA-MB-231 cell lines.^2^ Due to its biological activity, cananodine is the most prominent member of the guaipyridine alkaloids, which include the eponymous compound and the rupestines (Figure 1). The rupestines were isolated from the flowers and leaves of *Artemisia rupestris,* which is used in traditional Chinese medicine.^3^ The guaipyridines have a methyl group at C5 but varying substituents at C8 of the bicyclic core.

Guaipyridine alkaloids have long attracted the attention of synthetic chemists. Early studies by van der Gen^4^ and Okatani^5^ are best described as stereo- and regiorandom, yielding mixtures of constitutional isomers in the cyclization reaction to form the seven-membered ring. In 2006, Craig and Henry synthesized (+)-cananodine from (*R*)-(−)-citronellene and utilized a clever microwave-assisted decarboxylative Claisen rearrangement as the key step.^6^ In 2017, we reported the synthesis of all four stereoisomers of cananodine using the opening of a trisubstituted epoxide to construct the seven-membered ring of the target.^7^ We subsequently synthesized (±)-cananodine, (±)-rupestine G, and (±)-rupestine D using an intramolecular Mizoroki–Heck reaction to form the seven-membered carbocycle skeleton of the natural products.^8^ More recently, the Aisa group prepared (−)-cananodine and (−)-rupestine D from (*S*)-(+)-citronellene.^9^ Finally, the Yusuf group made (±)-cananodine and separated the enantiomers using chiral HPLC.^2^ Herein, we report the synthesis of (+)- and (−)-cananodine using an Evans alkylation to set the C8 stereocenter. There are a number of values given in the literature^1,2,6,9^ for the optical rotation of cananodine, and our work making both enantiomers in sufficient quantities settles any dispute over optical rotation of the natural product. Making both mirror images also allows for further biological investigation of each enantiomer.

**Scheme 1:**
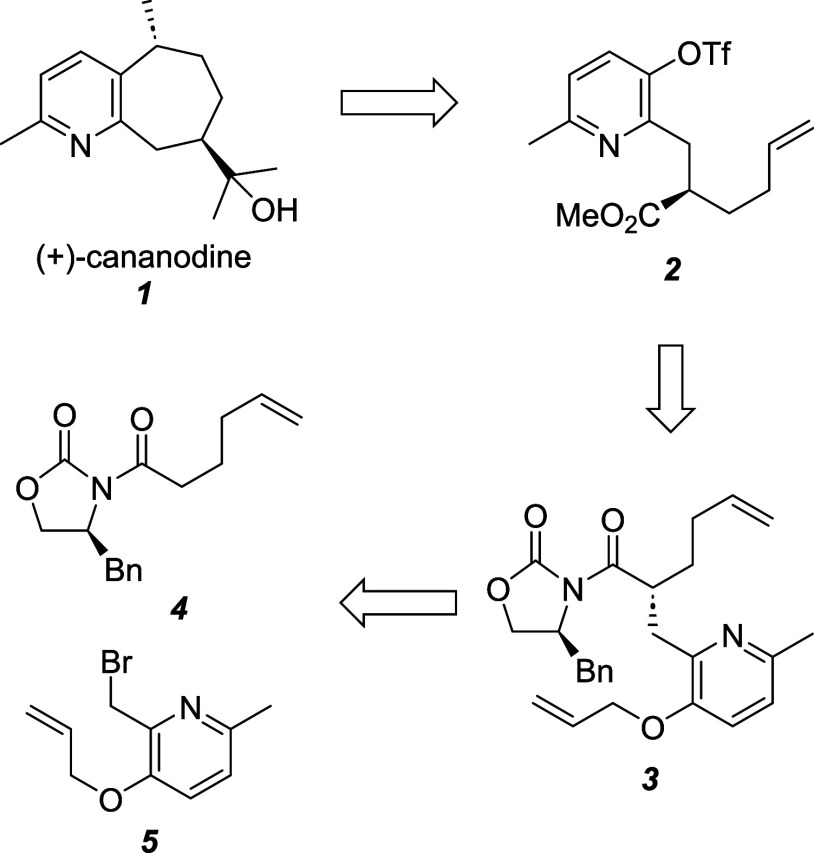
Retrosynthetic Analysis of Cananodine

In our retrosynthetic analysis of (+)-cananodine (**1**), we envisaged preparing the seven-membered carbocycle through an intramolecular Mizoroki–Heck reaction of triflate **2** (Scheme 1). This triflate would, in turn, be made from **3**, the product of an Evans alkylation of oxazolidinone **4** with picolyl bromide **5**.

## Results and Discussion

**Scheme 2:**
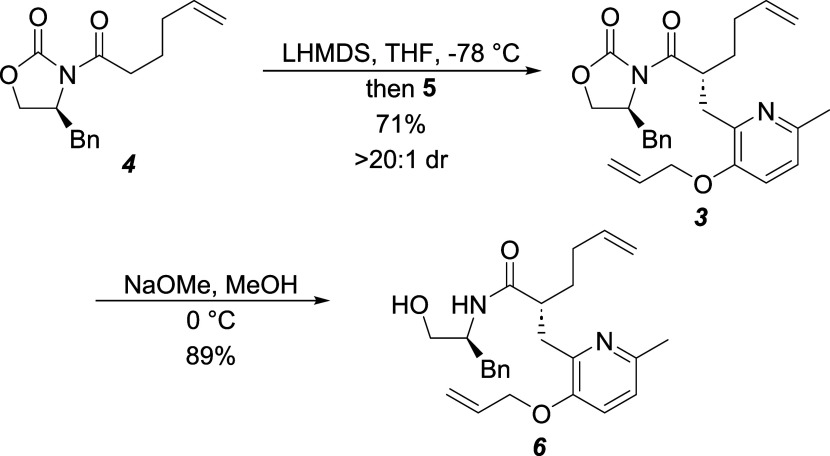
Alkylation and Attempted Auxiliary Cleavage

The synthesis of (+)-cananodine (**1**) commenced with the alkylation^10^ of oxazolidinone **4**(12) with picolyl bromide **5** (prepared in 3 steps from 6-methylpyridin-3-ol)^8,11^ to produce **3** in good yield and >96% diastereomeric excess (Scheme 2). We initially attempted this alkylation with the corresponding aryl triflate derivative of **5** instead of the allyl ether-protected phenol. The alkylation worked, but the purification of the product was exceedingly difficult, resulting in lower yields of pure material. Thus, we resorted to the allyl ether **5**, which did not have these separation difficulties.

**Scheme 3:**
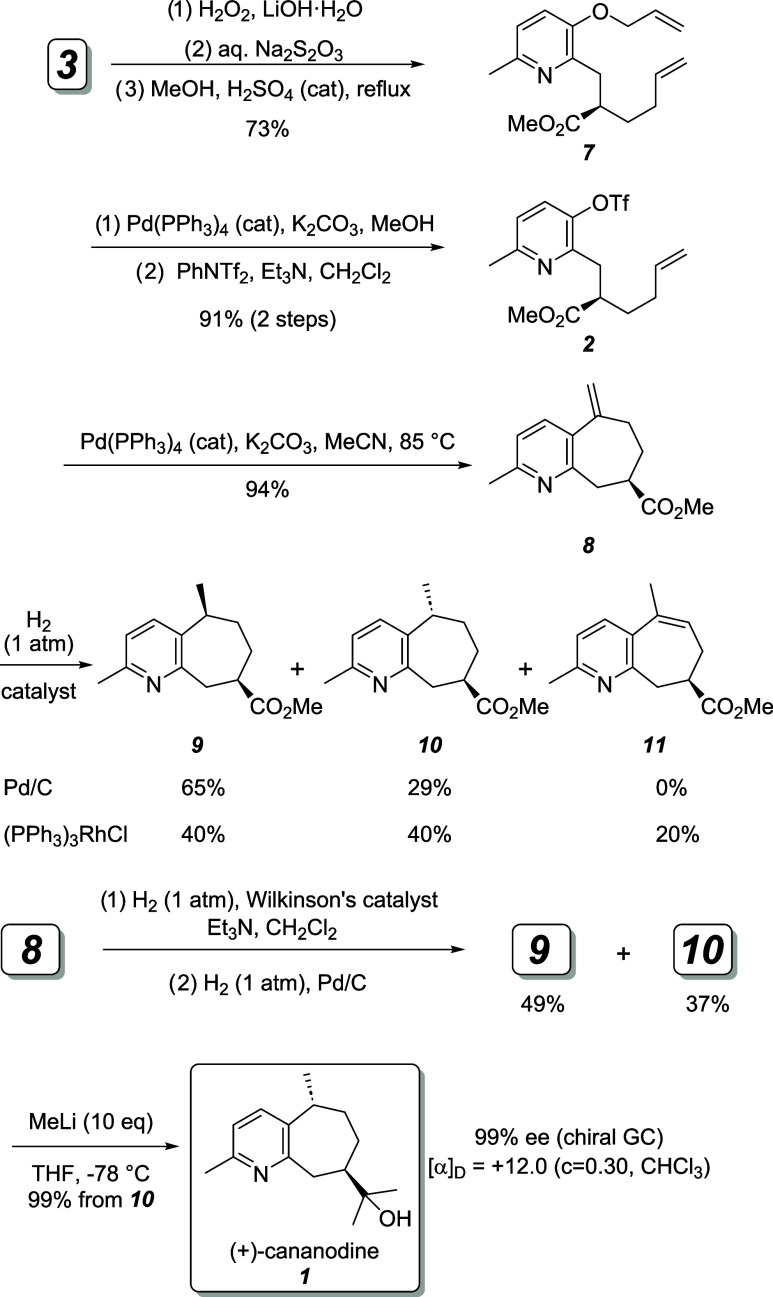
Synthesis of (+)-Cananodine (**1**)

Despite Craig and Henry’s report to the contrary on a similar oxazolidinone,^6^ treatment of **3** with methoxide did not produce the methyl ester, but rather hydroxyamide **6**.^13^ Standard cleavage of the chiral auxiliary with basic peroxide readily produced the corresponding carboxylic acid^10,13^ that was then subjected to Fischer esterification, which provided **7** in good yield and 99% enantiomeric excess, as determined by chiral GC analysis (Scheme 3). Deprotection of the allyl ether with catalytic Pd(PPh_3_)_4_ in basic methanol revealed the phenol, which was converted to the aryl triflate **2** without incident. The intramolecular Mizoroki–Heck reaction of **2** proceeded in excellent yield, provided that the Pd(PPh_3_)_4_ catalyst was washed with methanol immediately prior to the procedure to remove oxidized impurities, and provided bicyclic compound **8**.

The next task was to hydrogenate the exocyclic methylene of **8**. Consistent with prior results,^8^ heterogeneous hydrogenation with Pd on carbon gave a 2:1 mixture of undesired (+)-rupestine G (**9**) and the desired intermediate **10**. Utilizing Wilkinson’s catalyst for the hydrogenation improved the ratio of **9** to **10** to 1:1^7^ but also produced a significant amount of endocyclic alkene **11**, evidenced by a resonance at 6.0 ppm (ddq, *J* = 6.9, 6.9, 1.6 Hz) in the ^1^H NMR spectrum of the mixture.^14^ This isomer was resistant to reduction under one atm of hydrogen with Wilkinson’s catalyst. Use of Crabtree’s catalyst for the hydrogenation did not improve the diastereomeric ratio. Thus, we carried out a combination hydrogenation, first with Wilkinson’s catalyst, followed by treatment with hydrogen over palladium on carbon to produce **9** ((+)-rupestine G) and **10** in essentially a 1:1 ratio in high yield. Separation of the diastereomers and exhaustive methylation of ester **10** gave (+)-cananodine (**1**). Chiral GC analysis of the product showed a 99% ee, and the optical rotation [α]_D_ = +12.0, (*c* = 0.30 CHCl_3_) matched that measured by Yusuf et al. [α]_D_ = +10.0 (*c* = 0.06 CHCl_3_).^2^

**Figure 2:**
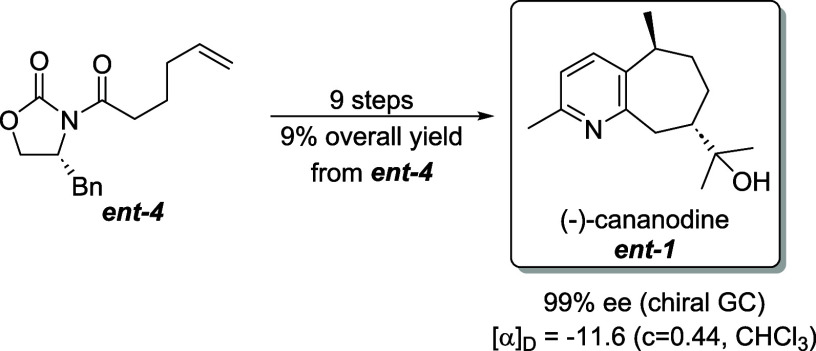
Synthesis of (−)-Cananodine (**2**).

Using the procedures optimized for the preparation of (+)-cananodine, the synthesis of (−)-cananodine was straightforward (Figure 2). Initiated by alkylation of oxazolidinone **ent-4**(15) with picolyl bromide **5**, and following the steps outlined in Schemes 2 and 3 provided (−)-cananodine (**ent-1**) in 99% ee by chiral GC analysis and an optical rotation that matched that previously reported: [α]_D_ = −11.6 (*c* = 0.44, CHCl_3_); lit^2^ [α]_D_ = −10.0, (*c* = 0.06, CHCl_3_).

## Conclusions

To summarize, we have accomplished the synthesis of both enantiomers of cananodine in nine steps and a 13% overall yield for (+)-cananodine (**1**) and a 9% yield for (−)-cananodine (**ent-1**). Key steps included a highly diastereoselective alkylation of the Evans auxiliary with picolyl bromide **5** and an intramolecular Mizoroki–Heck reaction to prepare the seven-membered ring of the target molecules. This efficient and scalable synthesis will enable further biological studies of cananodine and should facilitate the synthesis of structural analogues for biological testing.

## Experimental Section

All glassware was oven-dried and all reactions using air-sensitive materials were carried out under an argon atmosphere. Also, when indicated, dry solvent from the Inert PureSolv solvent purification system (Et_2_O, THF, CH_2_Cl_2_, CH_3_CN) was used. All solvents that were not from the purification system were HPLC grade and used without further purification, with the exception of MeOH, which was dried over 3A molecular sieves (8–12 mesh, Acros Organics) and CHCl_3_, which was flushed through basic alumina (Sorbtech, pH = 10). Celite (EMD Chemicals) containing diatomaceous earth, quartz, and cristobalite was not acid-washed during manufacturing.

Starting materials that were commercially available were used without further purification: 5-hexenoic acid (>98%, Tokyo Chemical Industry Co., LTD), 6-methyl-pyridin-3-ol (98%, Combi-Blocks), *N*-Phenyl-bis(trifluoromethanesulfonamide) (PhNTf_2_) (99%, Oakwood Chemical), (*S*)- and (*R*)-4-benzyl-2-oxazolidinone (98%, Combi-Blocks). All catalysts utilized were commercially available (Pd(PPh_3_)_4_ (99%, Strem Chemicals, lots L01412105 and L03342206), Pd/C, (10% Pd, Aldrich Chemical Co., lot 03803HP), and Wilkinson’s catalyst (99%, Strem Chemicals, lot # B0170086)). All were used without further purification besides Pd(PPh_3_)_4_, which was washed with methanol and dried by using vacuum filtration.

Each reaction involving extractive workup with the organic solvents and aqueous solutions detailed was washed with saturated NaCl (brine), dried over Na_2_SO_4_, and concentrated using rotary evaporation. All flash column chromatography was conducted using silica gel (230–400 mesh, Silicycle) hand-packed with varying ratios of hexanes and ethyl acetate (hexanes/EtOAc) unless otherwise indicated. Silica G TLC plates (Sorbtech, polyester backed, thickness 200 μM, fluorescence UV_254_) were used for monitoring the reaction progress and flash chromatography.

Infrared spectra (IR) were collected on a ThermoiS10 FT-IR spectrometer equipped with a single bounce diamond ATR. All tabulated signals are reported in cm^–1^. Spectra acquired as “neat” were placed on the diamond ATR stage as a pure solid or liquid or occasionally as films from pure compounds dissolved in CDCl_3_ or CH_2_Cl_2_ and then evaporated.

For NMR analysis, all samples were dissolved in deuterated chloroform (D-99.8%, +0.05% v/v TMS). ^1^H and ^13^C NMR spectra were acquired on a Varian MercuryPlus FT-NMR (300 MHz) or Bruker Avance III FT-NMR (500 MHz) instrument and processed with MestreNova software. Chemical shifts are reported in ppm, and coupling constants are reported in Hertz (Hz). ^1^H NMR spectra in CDCl_3_ are referenced to tetramethylsilane (TMS) at 0.00 ppm and reported using the format: chemical shift (ppm) [multiplicity (s = singlet, d = doublet, t = triplet, q = quartet, m = multiplet, app = apparent), coupling constant(s) (*J* in Hz), integral]. ^13^C NMR spectra are referenced to CDCl_3_ at 77.0 ppm.

Chiral gas chromatography (GC) was performed on a Varian CP3800 GC using an Agilent Cyclosil-B 30 m × 0.25 mm ID × 0.25 μm chiral column. All chiral samples were characterized using Method A. **Method A**: Hold 5 min at 60 °C, ramp 5 °C/min to 240 °C, hold 5 min.

Specific rotation was measured by using a Rudolph Digital Automatic polarimeter with a 10 cm quartz cell at room temperature (wavelength = 589 nm).

Mass spectrometry was conducted using an Agilent 6545XT AdvanceBio LC-ESI-qTOF mass spectrometer to acquire HRMS spectra. An Agilent ZORBAX Eclipse Plus C18 column was used (2.1 mm × 50 mm, 1.8 μm).

### (*S*)-3-((*R*)-2-((3-(Allyloxy)-6-methylpyridin-2-yl)methyl)hex-5-enoyl)-4-benzyloxazolidin-2-one (**3**)

Oxazolidinone **4** (0.829 g, 3.00 mmol) was placed under Ar. THF (10 mL) was added. This solution was cooled to −78 °C, and then LHMDS (3.60 mL, 1.0 M in THF, 3.60 mmol) was added dropwise. After stirring for three h, picolyl bromide **5** (0.726 g, 3.00 mmol) was dissolved in THF (15 mL) and added immediately. This was stirred in an ice bath warming slowly to room temperature overnight. The reaction was quenched with sat. NH_4_Cl and diluted in EtOAc and then extracted two times with EtOAc. The combined organic layers were washed twice with sat. NaCl and dried over Na_2_SO_4_. The crude product was purified using flash column chromatography (9:1 hexanes/acetone), and pure product **3** was isolated as a pale yellow oil (0.926 g, 2.13 mmol, 71%). **[α]**_**D**_^**20**^ + 36.2 (*c* 5.03, CHCl_3_). **Chiral GC:** Method A: Retention times: 42.40 min (major) and 43.95 min.(minor). 96% de. **IR:** 3063, 2927, 1782, 1695, 1454, 1387, 1257, 1189, 1102, 991, 911, 737, 700 cm^–1^. ^**1**^**H NMR** (500 MHz, CDCl_3_): δ 7.30 (app t, 2H), 7.25 (app t, 1H), 7.20 (app d, 2H), 6.96 (d, *J =* 8.4 Hz, 1H), 6.87 (d, *J =* 8.3 Hz, 1H), 6.06 (ddt, *J =* 17.2, 10.4, 5.2 Hz, 1H), 5.82 (dddd, *J =* 16.9, 10.2, 6.6, 6.6 Hz, 1H), 5.42 (dddd, *J =* 17.2, 1.6, 1.6, 1.6 Hz, 1H), 5.30 (dddd, *J =* 10.7, 1.7, 1.7, 1.7 Hz, 1H), 5.02 (dddd, *J =* 17.1, 1.7, 1.7, 1.7 Hz, 1H), 4.95 (app dd, 1H), 4.63 (dddd, *J =* 10.2, 6.4, 3.2, 3.2 Hz, 2H), 4.53 (ddd, *J =* 5.2, 1.6, 1.6 Hz, 2H), 4.50 (dddd, *J =* 11.0, 6.8, 6.8, 4.3 Hz, 1H), 4.14–4.08 (m, 2H), 3.35 (dd, *J =* 13.3, 3.2 Hz, 1H), 3.31 (dd, *J =* 15.6, 10.1 Hz, 1H), 3.16 (dd, *J =* 15.6, 4.3 Hz, 1H), 2.53 (dd, *J =* 13.3, 10.6 Hz, 1H), 2.38 (s, 3H), 2.25–2.13 (m, 2H), 1.88 (dddd, *J =* 13.4, 9.9, 6.6, 6.6 Hz, 1H), 1.73 (dddd, *J =* 13.6, 9.3, 6.4, 6.4 Hz, 1H) ppm. ^**13**^**C{**^**1**^**H} NMR** (125 MHz, CDCl_3_): δ 176.7, 152.9, 150.7, 148.7, 148.4, 138.3, 136.1, 133.2, 129.4, 128.9, 127.1, 121.0, 118.4, 117.8, 114.8, 69.0, 65.8, 55.9, 40.2, 37.9, 34.3, 32.0, 31.4, 23.5 ppm. **HRMS:** (ESI, q-TOF) *m*/*z* [M + H] calcd for C_26_H_30_N_2_O_4_ 435.2278; found 435.2280.

### (*R*)-2-((3-(Allyloxy)-6-methylpyridin-2-yl)methyl)-*N*-((*S*)-1-hydroxy-3-phenylpropan-2-yl)hex-5-enamide (**6**)

NaOMe (0.131 g, 2.43 mmol) was put under an Ar atmosphere and then dissolved in MeOH (5 mL), and the solution was cooled in an ice bath. Oxazolidinone **3** (1.057 g, 2.428 mmol) was added, and the mixture was stirred for 30 min. At this time, H_2_O (20 mL) and sat. NH_4_Cl (15 mL) were added, and the solution was extracted three times using CH_2_Cl_2_. The combined organic layers were dried over Na_2_SO_4_, and the solvent was removed. The crude product was purified using flash column chromatography (2:1 hexanes/EtOAc), and hydroxyamide **6** was isolated as an off-white solid (0.885 g, 2.16 mmol, 89%). **[α]**_**D**_^**20**^ – 66.5 (*c* 1.53, CHCl_3_). **IR:** 3267, 3022, 2924, 2859, 2100, 1649, 1458, 1259, 1054, 695 cm^–1^. ^**1**^**H NMR** (500 MHz, CDCl_3_): δ 7.24 (app t, 2H), 7.18 (app t, 1H), 7.16 (app d, 2H), 7.01 (d, *J =* 8.4 Hz, 1H), 6.92 (d, *J =* 8.4 Hz, 1H), 6.53 (d, *J =* 7.8 Hz, 1H), 6.01 (ddt, *J =* 17.4, 15.7, 5.2 Hz, 1H), 5.75 (dddd, *J =* 16.9, 10.2, 6.7, 6.7 Hz, 1H), 5.39 (dddd, *J =* 17.2, 1.7, 1.7, 1.7 Hz, 1H), 5.29 (dddd, *J =* 10.5, 1.5, 1.5, 1.5 Hz, 1H), 4.98 (app dd, 1H), 4.93 (app d, 1H), 4.51 (app d, 2H), 4.09 (m, 1H), 3.66 (dd, *J =* 11.3, 3.4 Hz, 1H), 3.49 (dd, *J =* 11.3, 4.6 Hz, 1H), 3.08 (dd, *J =* 14.2, 3.7 Hz, 1H), 2.85 (m, 1H), 2.80 (app d, 2H), 2.43 (s, 3H), 2.03 (app q, 2H), 1.78 (m, 2H), 1.55 (m, 2H) ppm. ^**13**^**C{**^**1**^**H} NMR** (125 MHz, CDCl_3_): δ 175.6, 150.8, 148.6, 148.5, 138.2, 138.0, 132.7, 129.1, 128.5, 126.4, 121.8, 119.4, 117.8, 114.8, 69.0, 63.5, 52.7, 45.2, 37.1, 35.4, 32.0, 31.4, 22.9 ppm. **HRMS** (ESI, q-TOF) *m*/*z* [M + H] calcd for C_25_H_32_N_2_O_3_ 409.2486; found 409.2491.

### Methyl (*R*)-2-((3-(Allyloxy)-6-methylpyridin-2-yl)methyl)hex-5-enoate (**7**)

LiOH·H_2_O (0.340 g, 8.10 mmol) was placed in a flask, and H_2_O (2 mL) was added. This was cooled in an ice bath, H_2_O_2_ (30%, 1.84 mL, 23 mmol) was added, and the solution was stirred for 15 min. Alkylated oxazolidinone **3** (1.135 g, 2.607 mmol) was dissolved in THF (8 mL, final ratio 4:1, THF: H_2_O) and added to the mixture. The reaction was stirred for 3 h and deemed complete by TLC analysis. Na_2_S_2_O_3_ solution was used to quench the excess peroxides, stirring at 0 °C for 15 min. The solution was acidified to pH = 6 with NH_4_Cl, and the product was extracted with EtOAc three times. The combined organic layers were washed with sat. NaCl and dried over Na_2_SO_4_. The solvent was removed by using rotary evaporation. The crude acid product was dissolved in MeOH (10 mL), and 8 drops of conc. H_2_SO_4_ was added. The reaction mixture was stirred and refluxed overnight. After 24 h, the reaction was complete by TLC. The solvent was removed, and pH was neutralized using NaHCO_3_ after dilution in EtOAc, which was used to extract the product from the aqueous layer three times. The combined organic layers were washed once with sat. NaCl and then dried over Na_2_SO_4_. After the solvent was removed, the crude ester was purified using flash column chromatography using 2:1 hexanes/EtOAc. Pure ester **7** was isolated as a clear, yellow oil (0.514 g, 1.78 mmol, 68%). **[α]**_**D**_^**20**^ – 9.7 (*c* 4.27, CHCl_3_). **Chiral GC Analysis:** Method A: Retention times: 37.60 min (minor) and 37.62 min (major) 99% ee. **IR:** 3082, 2994, 2930, 2849, 1731, 1458, 1255, 1161, 991, 916, 813 cm^–1^. ^**1**^**H NMR** (500 MHz, CDCl_3_): δ 7.01 (d, *J =* 8.3 Hz, 1H), 6.93 (d, *J =* 8.3 Hz, 1H), 6.05 (ddt, *J =* 17.3, 10.4, 5.5 Hz, 1H), 5.80 (dddd, *J =* 16.9, 10.2, 6.6, 6.6 Hz, 1H), 5.43 (dddd, *J =* 17.3, 1.7, 1.7, 1.7 Hz, 1H), 5.32 (dddd, *J =* 10.6, 1.5, 1.5, 1.5 Hz, 1H), 5.00 (dddd, *J =* 17.1, 1.6, 1.6, 1.6 Hz, 1H), 4.95 (dddd, *J =* 10.2, 1.3, 1.3, 1.3 Hz, 1H), 4.53 (ddd, *J =* 5.0, 1.6, 1.6 Hz, 2H), 3.66 (s, 3H), 3.15 (dd, *J =* 14.2, 8.2 Hz, 1H), 3.05 (dd, *J =* 14.2, 6.3 Hz, 1H), 3.00 (dddd, *J =* 8.6, 8.6, 6.4, 6.4 Hz, 1H), 2.45 (s, 3H), 2.17–2.03 (m, 2H), 1.83 (dddd, *J =* 14.9, 9.1, 9.1, 6.0 Hz, 1H), 1.64 (dddd, *J =* 11.5, 9.6, 6.3, 5.1 Hz, 1H) ppm. ^**13**^**C{**^**1**^**H} NMR** (125 MHz, CDCl_3_): δ 176.4, 150.5, 148.9, 148.4, 138.1, 132.9, 121.2, 118.8, 117.5, 114.8, 68.9, 51.3, 43.6, 34.5, 31.5, 31.2, 23.3 ppm. **HRMS** (ESI, q-TOF) *m*/*z* [M + H] calcd for C_17_H_23_NO_3_ 290.1751; found 290.1750.

### Methyl (*R*)-2-((6-Methyl-3-(((trifluoromethyl)sulfonyl)oxy)pyridin-2-yl)methyl)hex-5-enoate (**2**)

Pd(PPh_3_)_4_ (0.015 g, 0.013 mmol) was added to a flask containing pure ester **7** (0.376 g, 1.30 mmol) and K_2_CO_3_ (0.538 g, 3.89 mmol). After establishing an Ar atmosphere, MeOH (4 mL) was added, and the suspension was stirred overnight at room temperature. The mixture was then filtered over Celite and washed with EtOAc, followed by solvent removal. After diluting in CH_2_Cl_2_, NH_4_Cl was added to neutralize pH. The aqueous layer was extracted three times with CH_2_Cl_2_, and then the combined organic layers were washed with sat. NaCl. The solvent was removed to give the crude phenol as a hazy orange oil (0.355 g), which was used without purification. The crude phenol was added to a flask containing PhNTf_2_ (0.794 g, 2.22 mmol) and put under Ar. Dry CH_2_Cl_2_ (5 mL) was added and stirred before NEt_3_ (0.31, 2.2 mmol) was added. This mixture was stirred at room temperature overnight. Once deemed complete by TLC, the dark, cloudy blue solution was washed with 10% NaOH, sat. NH_4_Cl, and sat. NaCl, sequentially. This product was dried over Na_2_SO_4_, and the solvent was removed to give a thick blue oil. The crude product was purified by flash column chromatography using 6:1 hexanes/EtOAc, providing triflate **2** as a clear, colorless oil (0.442 g, 1.16 mmol, 89%). **[α]**_**D**_^**20**^ + 1.7 (*c* 0.29, CHCl_3_). **Chiral GC:** Method A: Retention times: 34.14 min (major) and 34.28 min (minor) 97% ee. **IR:** 3076, 2961, 2848, 1739, 1427, 1259, 1216, 1012, 794 cm^–1^. ^**1**^**H NMR** (500 MHz, CDCl_3_): δ 7.43 (d, *J =* 8.4 Hz, 1H), 7.05 (d, *J =* 8.4 Hz, 1H), 5.78 (dddd, *J =* 17.0, 10.3, 6.6, 6.6 Hz, 1H), 5.02 (app dd, 1H), 4.97 (app dd, 1H), 3.65 (s, 3H), 3.22 (dd, *J =* 14.8, 8.8 Hz, 1H), 3.09–3.04 (m, 1H), 3.00 (dd, *J =* 14.9, 5.1 Hz, 1H), 2.51 (s, 3H), 2.16–2.05 (m, 2H), 1.76 (app sextet, 1H), 1.67–1.60 (m, 1H) ppm. ^**13**^**C{**^**1**^**H} NMR** (125 MHz, CDCl_3_): δ 175.8, 158.1, 151.3, 143.3, 137.7, 129.0, 122.4, 118.5 (q, *J =* 320 Hz), 115.4, 51.7, 43.1, 33.9, 31.5, 31.4, 24.1 ppm. **HRMS** (ESI, q-TOF) *m*/*z* [M + H] calcd for C_15_H_18_F_3_NO_5_S 382.0931; found 382.0931.

### Methyl (*R*)-2-Methyl-5-methylene-6,7,8,9-tetrahydro-5*H*-cyclohepta[*b*] pyridine-8-carboxylate (**8**)

In a double-necked flask, K_2_CO_3_ (0.300 g, 2.17 mmol) and Pd(PPh_3_)_4_ (5 mol %, 0.026 g, 0.023 mmol) were added and put under Ar. CH_3_CN (2 mL) was added and stirred. Triflate **2** (0.165 g, 0.433 mmol) was dissolved in CH_3_CN (2.5 mL) and added to the solution. The reaction was refluxed for 6.5 h when an additional 5 mol % catalyst was added (0.026 g of Pd(PPh_3_)_4_, 0.023 mmol) and continued to reflux for an additional 13 h. The reaction was cooled and filtered over silica and washed with EtOAc (15 mL). The mixture was concentrated, and the crude product was purified using flash column chromatography (2:1 hexanes/EtOAc, *R*_f_ = 0.25). Bicyclic ester **8** was isolated as a clear, colorless oil (0.094 g, 0.41 mmol, 94%). **[α]**_**D**_^**20**^ – 71.2 (*c* 0.94, CHCl_3_). **Chiral GC:** Method A: Retention times: 34.87 min (major) and 35.04 min (minor) 98% ee. **IR:** 3073, 2943, 2855, 1730, 1590, 1435, 1165, 906, 828 cm^–1^. ^**1**^**H NMR** (500 MHz, CDCl_3_): δ 7.40 (d, *J =* 7.8 Hz, 1H), 6.98 (d, *J =* 7.8 Hz, 1H), 5.18 (app s, 1H), 5.07 (app s, 1H), 3.69 (s, 3H), 3.26 (dd, *J =* 14.6, 1.4 Hz, 1H), 3.17 (dd, *J =* 14.7, 10.5 Hz, 1H), 2.76 (dddd, *J =* 9.8, 9.8, 4.3, 2.7 Hz, 1H), 2.65 (ddd, *J =* 14.0, 7.6, 3.8 Hz, 1H), 2.51 (s, 3H), 2.33 (ddd, *J =* 13.9, 9.8, 4.0 Hz, 1H), 2.15 (app dq, 1H), 2.00 (dddd, *J =* 13.6, 9.8, 9.8, 3.8 Hz, 1H) ppm. ^**13**^**C{**^**1**^**H} NMR** (125 MHz, CDCl_3_): δ 175.7, 156.4, 156.0, 148.4, 136.0, 135.3, 121.3, 115.5, 51.8, 41.8, 40.5, 33.8, 32.9, 24.1 ppm. **HRMS:** (ESI, q-TOF) *m*/*z* [M + H] calcd for C_14_H_17_NO_2_ 232.1332; found 232.1336.

### Methyl (5*S*,8*R*)-2,5-Dimethyl-6,7,8,9-tetrahydro-5*H*-cyclohepta[*b*]pyridine-8-carboxylate (**9**) and Methyl (5*R*,8*R*)-2,5-Dimethyl-6,7,8,9-tetrahydro-5*H*-cyclohepta[*b*]pyridine-8-carboxylate (**10**)

To a flask containing bicyclic pyridine **8** (0.080 g, 0.35 mmol) was added Wilkinson’s catalyst (0.019 g, 0.021 mmol), and then the flask was put under an Ar atmosphere. Dry CH_2_Cl_2_ (3 mL) was added, followed by NEt_3_ (0.07 mL, 0.5 mmol). H_2_ gas (12” balloon) was flushed through the flask, and a second balloon was used to maintain an H_2_ atmosphere overnight. After the flask was purged with Ar, the mixture was filtered over silica using EtOAc to wash. The solvent was removed, leaving a brown, clear oil. The crude mixture of the two desired diastereomers and undesired endocycle were purified using flash column chromatography (1:1 hexanes/EtOAc) to give a mixture of both diastereomers and trace endocycle. Overlapping fractions were combined (0.048 g), and Pd/C (0.005 g, 10% w/w) was added and put under an Ar atmosphere before addition of MeOH (6 mL). H_2_ gas (12” balloon) was flushed through the flask, and a second balloon was used to maintain an H_2_ atmosphere overnight. The mixture was filtered over Celite and washed with EtOAc. The two products were purified using flash column chromatography (1:1 hexanes/EtOAc), and both **9** (0.040 g, 0.17 mmol) and **10** were isolated (0.030 g, 0.13 mmol) as clear, colorless oils (86% combined yield, 1.3:1 dr of **9** and **10**).

#### Methyl (5*S*,8*R*)-2,5-Dimethyl-6,7,8,9-tetrahydro-5*H*-cyclohepta[*b*]pyridine-8-carboxylate (**9**)

**[α]**_**D**_^**20**^ – 55.2 (*c* 0.27, CHCl_3_). **Chiral GC Analysis:** Method A: Retention times: 34.95 min (major) and 35.12 min (minor) 99% ee. **IR:** 2933, 2353, 1732, 1590, 1462, 1161 cm^–1^. ^**1**^**H NMR** (500 MHz, CDCl_3_): δ 7.30 (d, *J =* 7.8 Hz, 1H), 6.91 (d, *J =* 7.8 Hz, 1H), 3.61 (s, 3H), 3.36 (dd, *J =* 14.5, 9.7 Hz, 1H), 3.29 (dd, *J =* 14.7, 3.0 Hz, 1H), 2.99 (dddd, *J =* 14.3, 7.2, 7.2, 3.4 Hz, 1H), 2.65 (dddd, *J =* 9.6, 9.6, 3.2, 3.2 Hz, 1H), 2.48 (s, 3H) 2.12 (dddd, *J =* 14.0, 10.7, 9.4, 3.2 Hz, 1H) 1.98 (ddq, *J =* 14.0, 10.4, 3.5 Hz, 1H), 1.85 (dddd, *J =* 14.0, 6.9, 6.9, 3.2 Hz, 1H), 1.76 (dddd, *J =* 14.0, 10.7, 3.4, 3.4 Hz, 1H), 1.32 (d, *J =* 7.3 Hz, 3H) ppm. ^**13**^**C{**^**1**^**H} NMR** (125 MHz, CDCl_3_): δ 175.9, 157.6, 154.9, 137.9, 136.2, 121.4, 51.4, 42.2, 40.8, 37.8, 32.4, 29.2, 24.0, 18.9 ppm. **HRMS** (ESI, q-TOF) *m*/*z* [M + H] calcd for C_14_H_19_NO_2_ 234.1489; found 234.1489.

#### Methyl (5*R*,8*R*)-2,5-Dimethyl-6,7,8,9-tetrahydro-5*H*-cyclohepta[*b*]pyridine-8-carboxylate (**10**)

**[α]**_**D**_^**20**^ – 47.8 (*c* 0.09, CHCl_3_). **Chiral GC:** Method A: Retention times: 35.76 min (minor) and 35.92 min (major) 99% ee. **IR:** 2930, 2851, 1734, 1464, 1433, 1159, 731 cm^–1^. ^**1**^**H NMR** (500 MHz, CDCl_3_): δ 7.37 (d, *J =* 8.0 Hz, 1H), 6.98 (d, *J =* 7.8 Hz, 1H), 3.69 (s, 3H), 3.31 (dd, *J =* 14.0, 10.5 Hz, 1H), 3.25 (app d, 1H), 2.99 (app quin, 1H), 2.49 (s, 3H), 2.47 (dddd, *J =* 10.8, 10.8, 3.0, 3.0 1H), 2.18–2.12 (m, 1H), 1.97 (dddd, *J =* 11.8, 11.8, 11.8, 3.7 Hz, 1H), 1.88 (dddd, *J =* 14.0, 5.3, 3.8, 1.8 Hz, 1H), 1.34 (d, *J =* 7.0 Hz, 3H), 1.31–1.23 (m, 1H) ppm. ^**13**^**C NMR{**^**1**^**H}** (125 MHz, CDCl_3_): δ 176.2, 158.9, 154.5, 137.8, 132.4, 121.2, 51.8, 42.1, 40.6, 35.0, 34.8, 33.9, 23.9, 20.4 ppm. **HRMS** (ESI, q-TOF) *m*/*z* [M + H] calcd for C_14_H_19_NO_2_ 234.1489; found 234.1488.

### 2-((5*R*,8*R*)-2,5-Dimethyl-6,7,8,9-tetrahydro-5*H*-cyclohepta[*b*]pyridin-8-yl)propan-2-ol [(+)-cananodine] (**1**)

Ester **10** (0.057 g, 0.24 mmol) was put under Ar, and then THF (5 mL) was added and cooled to −78 °C. MeLi (3.1 M in diethoxymethane, 0.80 mL, 2.60 mmol) was added dropwise, and the mixture was stirred for 15 min. The reaction was removed from the dry ice bath and deemed complete by TLC (EtOAc) after 15 additional minutes. Et_2_O (20 mL) was added, and the reaction was quenched with sat. NH_4_Cl. The organic layer was washed once with sat. NaCl and then dried over Na_2_SO_4_. The solvent was removed, leaving product (**1**) as a clear, yellow gel (0.056 g, 0.24 mmol, 99%). **[α]**_**D**_^**20**^ + 12.0 (*c* 0.30, CHCl_3_). **Chiral GC:** Method A: Retention times: 36.54 min (minor) and 36.66 min (major). 99% ee. **IR:** 3364, 2967, 2913, 2869, 1590, 1459, 1145, 920, 731 cm^–1^. ^**1**^**H NMR** (500 MHz, CDCl_3_): δ 7.34 (d, *J =* 8.0 Hz, 1H), 6.93 (d, *J =* 8.0 Hz, 1H), 3.21 (app d, 1H), 2.97 (app quin 1H), 2.88 (dd, *J =* 13.4, 10.4 Hz, 1H), 2.48 (s, 3H), 2.14–2.08 (m, 1H), 1.89 (ddq, *J =* 13.7, 6.5, 4.9 Hz, 1H), 1.60 (dddd, *J =* 12.1, 12.1, 12.1, 3.6 Hz, 1H), 1.42 (dddd, *J =* 11.8, 10.3, 2.9, 1.5 Hz, 1H), 1.32 (d, *J =* 7.0 Hz, 3H), 1.26 (s, 3H), 1.25 (s, 3H), 1.30–1.21 (m, 1H) ppm. ^**13**^**C NMR** (125 MHz, CDCl_3_): δ 160.9, 154.3, 137.9, 132.4, 120.6, 73.3, 48.0, 39.8, 36.1, 35.3, 32.6, 27.6, 26.0, 23.9, 20.7 ppm. **HRMS** (ESI, q-TOF) *m*/*z* [M + H] calcd for C_15_H_23_NO 234.1852; found 234.1858.

## Supporting Information Available

Detailed experimental procedures, characterization data, and copies of ^1^H and ^13^C{^1^H} NMR spectra (PDF)
