# Supplementary material for: Enantioselective Synthesis of the Guaipyridine Alkaloid (+)- and (−)-Cananodine
Source: ACS Omega. 2024 Feb 7;9(7):7904–9. doi: 10.1021/acsomega.3c07735 (PMC10882590; doi:10.1021/acsomega.3c07735)
Supplement: Supplementary file 1 — ao3c07735_si_001.pdf [file ao3c07735_si_001.pdf]

Supporting Information for

Enantioselective Synthesis of the Guaipyridine Alkaloid (+)- and (-)-Cananodine

Haley M. Holliday, Kendelyn I. Bone, Rhemrose Sabio, and James R. Vyvyan*

Department of Chemistry, Western Washington University, 516 High Street, Bellingham, WA 98225-9150

vyvyan@wwu.edu

Contents

General Experimental Procedures S2

Experimental Procedures S4

Copies of ^{1}H and ^{13}C NMR Spectra S17

Experimental

All glassware was oven-dried and all reactions using air sensitive materials was carried out under argon atmosphere. Also, when indicated, dry solvent from Inert PureSolv™ Solvent Purification System (Et_2O, THF, CH_2Cl_2, CH_3CN) was used. All solvents that were not from the purification system was HPLC grade and used without further purification with the exception of MeOH which was dried over 3A molecular sieves (8-12 mesh, Acros Organics) and $CHCl_3$ which was flushed through basic alumina (Sorbtech, pH = 10). Celite (EMD Chemicals) containing diatomaceous earth, quartz, and cristobalite was not acid washed during manufacturing.

Starting materials that were commercially available were used without further purification: 5-hexenoic acid (>98%, Tokyo Chemical Industry Co., LTD), 6-methyl-pyridin-3-ol (98%, Combi-Blocks), N-Phenyl-bis(trifluoromethanesulfonamide ($PhNTf_2$) (99%, Oakwood Chemical), (*S*)- and (*R*)-4-benzyl-2-oxazolidinone (98%, Combi-Blocks). All catalysts utilized were commercially available ($Pd(PPh_3)_4$ (99%, Strem Chemicals, lot # L01412105 and # L03342206), Pd/C, (10% Pd, Aldrich Chemical Co., lot # 03803HP) and Wilkinson's catalyst (99%, Strem Chemicals, lot # B0170086)). All were used without further purification besides $Pd(PPh_3)_4$ which was washed with methanol and dried using vacuum filtration.

Each reaction involving extractive work-up with the organic solvents and aqueous solutions detailed was washed with saturated NaCl (brine), dried over Na_2SO_4, and concentrated using rotary evaporation. All flash column chromatography was conducted using silica gel (230-400 mesh, Silicycle) hand-packed with varying ratios of hexanes and ethyl acetate (hexanes:EtOAc) unless otherwise indicated. Silica G TLC plates (Sorbtech, polyester backed, thickness 200 μM, fluorescence UV_{254}) were used for monitoring reaction progress and flash chromatography.

Infrared spectra (IR) were collected on a ThermoiS10 FT-IR spectrometer equipped with a single bounce diamond ATR. All tabulated signals are reported in cm^{-1}. Spectra acquired as 'neat' were placed on the diamond ATR stage as a pure solid or liquid, or occasionally as films from pure compounds dissolved in $CDCl_3$ or CH_2Cl_2 then evaporated.

For NMR analysis, all samples were dissolved in deuterated chloroform (D-99.8%, +0.05% v/v TMS). ^1H and ^{13}C NMR spectra were acquired on a Varian MercuryPlus FT-NMR spectrometer (300 MHz) or Bruker Avance III FT-NMR spectrometer (500 MHz) and processed with MestreNova software. Chemical shifts are reported in ppm and coupling constants are reported in Hertz (Hz). ^1H NMR spectra in $CDCl_3$ are referenced to tetramethylsilane (TMS) at 0.00 ppm and reported using the format: chemical shift (ppm) [multiplicity (s = singlet, d = doublet, t = triplet, q = quartet, m = multiplet, app = apparent), coupling constant(s) (*J* in Hz), integral]. ^{13}C NMR spectra are referenced to $CDCl_3$ at 77.0 ppm.

Chiral gas chromatography (GC) was performed on a Varian CP3800 GC using an Agilent Cyclosil-B 30 m × 0.25 mm ID × 0.25 μm chiral column. All chiral samples were characterized using Method A.

Method A: Hold 5 minutes at 60 °C, ramp 5 °C/min to 240 °C, hold 5 minutes.

Specific rotation was measured using a Rudolph Digital Automatic polarimeter with a 10 cm quartz cell at room temperature (wavelength = 589 nm).

Mass spectrometry was conducted using an Agilent 6545XT AdvanceBio LC-ESI-qTOF mass spectrometer to acquire HRMS spectra. An Agilent ZORBAX Eclipse Plus C18 column was used (2.1 x 50 mm, 1.8 µm). All samples were dissolved and diluted in CH_3CN (3-10 µg/mL) and eluted using a gradient of **A** (water, 0.1% formic acid) and **B** (CH_3CN, 0.1% formic acid). Sample injection volume was between 0.1-0.01 µL. Flow rate was 0.400 mL/min and maximum pressure was set to 1,100.00 bar. All samples were characterized using Method B.

Method B:

**Time**	**A (%)**	***B (%)***
0.00	95.00	5.00
1.00	95.00	5.00
6.00	5.00	95.00
6.50	5.00	95.00
6.60	95.00	5.00
8.00	90.00	10.00

(*S*)-3-((*R*)-2-((3-(Allyloxy)-6-methylpyridin-2-yl)methyl)hex-5-enoyl)-4-benzyloxazolidin-2-one (3)

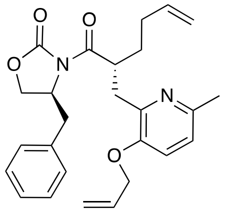

Oxazolidinone **4** (0.829 g, 3.00 mmol) was put under Ar. THF (10 mL) was added. This solution was cooled to -78 °C, then LHMDS (3.60 mL, 1.0 M in THF, 3.60 mmol) was added dropwise. After stirring for three hours, picolyl bromide **5** (0.726 g, 3.00 mmol) was dissolved in THF (15 mL) and added immediately. This was stirred in an ice bath warming slowly to room temperature overnight. The reaction was quenched with sat. NH_4Cl and diluted in EtOAc, and extracted two times with EtOAc. The combined organic layers were washed twice with sat. NaCl and dried over Na_2SO_4. The crude product was purified using flash column chromatography (9:1 hexanes:acetone) and pure product **3** was isolated as a pale yellow oil (0.926 g, 2.13 mmol, 71%).

$[\alpha]^{20}_D$ +36.2 (*c* 5.03, $CHCl_3$)

Chiral GC: Method A: Retention times: 42.40 min. (major) and 43.95 min.(minor). 96% de.

IR: 3063, 2927, 1782, 1695, 1454, 1387, 1257, 1189, 1102, 991, 911, 737, 700 cm^{-1}.

^{1}H NMR (500 MHz, $CDCl_3$): δ 7.30 (app t, 2H), 7.25 (app t, 1H), 7.20 (app d, 2H), 6.96 (d, *J* = 8.4 Hz, 1H), 6.87 (d, *J* = 8.3 Hz, 1H), 6.06 (ddt, *J* = 17.2, 10.4, 5.2 Hz, 1H), 5.82 (dddd, *J* = 16.9, 10.2, 6.6, 6.6 Hz, 1H), 5.42 (dddd, *J* = 17.2, 1.6, 1.6, 1.6 Hz, 1H), 5.30 (dddd, *J* = 10.7, 1.7, 1.7, 1.7 Hz, 1H), 5.02 (dddd, *J* = 17.1, 1.7, 1.7, 1.7 Hz, 1H), 4.95 (app dd, 1H), 4.63 (dddd, *J* = 10.2, 6.4, 3.2, 3.2 Hz, 2H), 4.53 (ddd, *J* = 5.2, 1.6, 1.6 Hz, 2H), 4.50 (dddd, *J* = 11.0, 6.8, 6.8, 4.3 Hz, 1H), 4.14-4.08 (m, 2H), 3.35 (dd, *J* = 13.3, 3.2 Hz, 1H), 3.31 (dd, *J* = 15.6, 10.1 Hz, 1H), 3.16 (dd, *J* = 15.6, 4.3 Hz, 1H), 2.53 (dd, *J* = 13.3, 10.6 Hz, 1H), 2.38 (s, 3H), 2.25-2.13 (m, 2H), 1.88 (dddd, *J* = 13.4, 9.9, 6.6, 6.6 Hz, 1H), 1.73 (dddd, *J* = 13.6, 9.3, 6.4, 6.4 Hz, 1H) ppm.

^{13}C{^{1}H} NMR (125 MHz, $CDCl_3$): δ 176.7, 152.9, 150.7, 148.7, 148.4, 138.3, 136.1, 133.2, 129.4, 128.9, 127.1, 121.0, 118.4, 117.8, 114.8, 69.0, 65.8, 55.9, 40.2, 37.9, 34.3, 32.0, 31.4, 23.5 ppm.

HRMS: (ESI, q-TOF) *m/z* [M +H] calcd for $C_{26}H_{30}N_2O_4$ 435.2278; found 435.2280.

(*R*)-2-((3-(Allyloxy)-6-methylpyridin-2-yl)methyl)-*N*-((*S*)-1-hydroxy-3-phenylpropan-2-yl)hex-5-enamide (6)

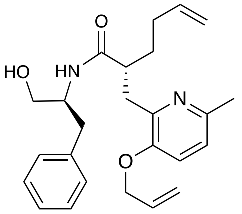

NaOMe (0.131 g, 2.43 mmol) was put under Ar atmosphere then dissolved in MeOH (5 mL) and the solution was cooled in an ice bath. Oxazolidinone **3** (1.057 g, 2.428 mmol) was added and the mixture was stirred for 30 minutes. At this time, H_2O (20 mL) and sat. NH_4Cl (15 mL) were added and the solution was extracted three times using CH_2Cl_2. The combined organic layers were dried over Na_2SO_4 and solvent was removed. The crude product was purified using flash column chromatography (2:1 hexanes:EtOAc) and hydroxyamide **6** was isolated as an off-white solid (0.885 g, 2.16 mmol, 89%).

$[\alpha]^{20}_{D}$ -66.5 (*c* 1.53, $CHCl_3$)

IR: 3267, 3022, 2924, 2859, 2100, 1649, 1458, 1259, 1054, 695 cm^{-1}.

^{1}H NMR (500 MHz, $CDCl_3$): *δ* 7.24 (app t, 2H), 7.18 (app t, 1H), 7.16 (app d, 2H), 7.01 (d, *J* = 8.4 Hz, 1H), 6.92 (d, *J* = 8.4 Hz, 1H), 6.53 (d, *J* = 7.8 Hz, 1H), 6.01 (ddt, *J* = 17.4, 15.7, 5.2 Hz, 1H), 5.75 (dddd, *J* = 16.9, 10.2, 6.7, 6.7 Hz, 1H), 5.39 (dddd, *J* = 17.2, 1.7, 1.7, 1.7 Hz, 1H), 5.29 (dddd, *J* = 10.5, 1.5, 1.5, 1.5 Hz, 1H), 4.98 (app dd, 1H), 4.93 (app d, 1H), 4.51 (app d, 2H), 4.09 (m, 1H), 3.66 (dd, *J* = 11.3, 3.4 Hz, 1H), 3.49 (dd, *J* = 11.3, 4.6 Hz, 1H), 3.08 (dd, *J* = 14.2, 3.7 Hz, 1H), 2.85 (m, 1H), 2.80 (app d, 2H), 2.43 (s, 3H), 2.03 (app q, 2H), 1.78 (m, 2H), 1.55 (m, 2H) ppm.

^{13}C{^{1}H} NMR (125 MHz, $CDCl_3$): *δ* 175.6, 150.8, 148.6, 148.5, 138.2, 138.0, 132.7, 129.1, 128.5, 126.4, 121.8, 119.4, 117.8, 114.8, 69.0, 63.5, 52.7, 45.2, 37.1, 35.4, 32.0, 31.4, 22.9 ppm.

HRMS (ESI, q-TOF) *m/z* [M +H] calcd for $C_{25}H_{32}N_2O_3$ 409.2486; found 409.2491.

Methyl (*R*)-2-((3-(allyloxy)-6-methylpyridin-2-yl)methyl)hex-5-enoate (7)

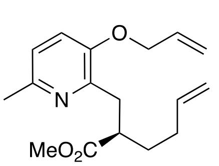

LiOH•H_2O (0.340 g, 8.10 mmol) was placed in a flask and H_2O (2 mL) was added. This was cooled in an ice bath and H_2O_2 (30%, 1.84 mL, 23 mmol) was added and the solution stirred for 15 minutes. Alkylated oxazolidinone **3** (1.135 g, 2.607 mmol) was dissolved in THF (8 mL, final ratio 4:1, THF: H_2O) then added to the mixture. The reaction was stirred for 3 hours and deemed complete by TLC analysis. $Na_2S_2O_3$ solution was used to quench the excess peroxides, stirring at

0 °C for 15 minutes. The solution was acidified to pH = 6 with NH_4Cl and product was extracted with EtOAc three times. The combined organic layers were washed with sat. NaCl and dried over Na_2SO_4. Solvent was removed using rotary evaporation. The crude acid product was dissolved in MeOH (10 mL) and 8 drops of conc. H_2SO_4 was added. The reaction was stirred and refluxed overnight. After 24 hours, the reaction was complete by TLC. Solvent was removed and pH was neutralized using $NaHCO_3$ after dilution in EtOAc which was used to extract the product from the aqueous layer three times. The combined organic layers were washed once with sat. NaCl and then dried over Na_2SO_4. After solvent was removed, the crude ester was purified using flash column chromatography using 2:1 hexanes:EtOAc. Pure ester **7** was isolated as a clear, yellow oil (0.514 g, 1.78 mmol, 68%).

$[\alpha]^{20}_D$ -9.7 (*c* 4.27, $CHCl_3$)

Chiral GC Analysis: Method A: Retention times: 37.60 min. (minor) and 37.62 min. (major) 99% ee.

IR: 3082, 2994, 2930, 2849, 1731, 1458, 1255, 1161, 991, 916, 813 cm^{-1}.

^{1}H NMR (500 MHz, $CDCl_3$): δ 7.01 (d, *J* = 8.3 Hz, 1H), 6.93 (d, *J* = 8.3 Hz, 1H), 6.05 (ddt, *J* = 17.3, 10.4, 5.5 Hz, 1H), 5.80 (dddd, *J* = 16.9, 10.2, 6.6, 6.6 Hz, 1H), 5.43 (dddd, *J* = 17.3, 1.7, 1.7, 1.7 Hz, 1H), 5.32 (dddd, *J* = 10.6, 1.5, 1.5, 1.5 Hz, 1H), 5.00 (dddd, *J* = 17.1, 1.6, 1.6, 1.6 Hz, 1H), 4.95 (dddd, *J* = 10.2, 1.3, 1.3, 1.3 Hz, 1H), 4.53 (ddd, *J* = 5.0, 1.6, 1.6 Hz, 2H), 3.66 (s, 3H), 3.15 (dd, *J* = 14.2, 8.2 Hz, 1H), 3.05 (dd, *J* = 14.2, 6.3 Hz, 1H), 3.00 (dddd, *J* = 8.6, 8.6, 6.4, 6.4 Hz, 1H), 2.45 (s, 3H), 2.17-2.03 (m, 2H), 1.83 (dddd, *J* = 14.9, 9.1, 9.1, 6.0 Hz, 1H), 1.64 (dddd, *J* = 11.5, 9.6, 6.3, 5.1 Hz, 1H) ppm.

^{13}C{^{1}H} NMR (125 MHz, $CDCl_3$): δ 176.4, 150.5, 148.9, 148.4, 138.1, 132.9, 121.2, 118.8, 117.5, 114.8, 68.9, 51.3, 43.6, 34.5, 31.5, 31.2, 23.3 ppm.

HRMS (ESI, q-TOF) *m/z* [M +H] calcd for $C_{17}H_{23}NO_3$ 290.1751; found 290.1750.

Methyl (*R*)-2-((6-methyl-3-(((trifluoromethyl)sulfonyl)oxy)pyridin-2-yl)methyl)hex-5-enoate (2)

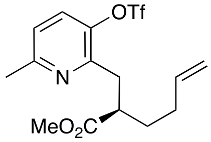

$Pd(PPh_3)_4$ (0.015 g, 0.013 mmol) was added to a flask containing pure ester **7** (0.376 g, 1.30 mmol) and K_2CO_3 (0.538 g, 3.89 mmol). After establishing Ar atmosphere, MeOH (4 mL) was added and the suspension was stirred overnight at room temperature. The mixture was then filtered over Celite washing with EtOAc, followed by solvent removal. After diluting in CH_2Cl_2, NH_4Cl was added to neutralize pH. The aqueous layer was extracted three times with CH_2Cl_2, then the combined organic layers were washed with sat. NaCl. The solvent was removed to give the crude phenol as a hazy orange oil (0.355 g) which was used without purification. The crude

phenol was added to a flask containing $PhNTf_2$ (0.794 g, 2.22 mmol) and put under Ar. Dry CH_2Cl_2 (5 mL) was added and stirred before NEt_3 (0.31, 2.2 mmol) was added. This mixture was left to stir at room temperature overnight. Once deemed complete by TLC, the dark, cloudy blue solution was washed with 10% NaOH, sat. NH_4Cl, and sat. NaCl sequentially. This product was dried over Na_2SO_4, and solvent was removed to give a thick blue oil. The crude product was purified by flash column chromatography using 6:1 hexanes:EtOAc, providing triflate **2** as a clear, colorless oil (0.442 g, 1.16 mmol, 89%).

$[\alpha]^{20}_D$ +1.7 (*c* 0.29, $CHCl_3$)

Chiral GC: Method A: Retention times: 34.14 min. (major) and 34.28 min. (minor) 97% ee.

IR: 3076, 2961, 2848, 1739, 1427, 1259, 1216, 1012, 794 cm^{-1}.

^{1}H NMR (500 MHz, $CDCl_3$): δ 7.43 (d, J = 8.4 Hz, 1H), 7.05 (d, J = 8.4 Hz, 1H), 5.78 (dddd, J = 17.0, 10.3, 6.6, 6.6 Hz, 1H), 5.02 (app dd, 1H), 4.97 (app dd, 1H), 3.65 (s, 3H), 3.22 (dd, J = 14.8, 8.8 Hz, 1H), 3.09-3.04 (m, 1H), 3.00 (dd, J = 14.9, 5.1 Hz, 1H), 2.51 (s, 3H), 2.16-2.05 (m, 2H), 1.76 (app sextet, 1H), 1.67-1.60 (m, 1H) ppm.

^{13}C{^{1}H} NMR (125 MHz, $CDCl_3$): δ 175.8, 158.1, 151.3, 143.3, 137.7, 129.0, 122.4, 118.5 (q, J = 320 Hz), 115.4, 51.7, 43.1, 33.9, 31.5, 31.4, 24.1 ppm.

HRMS (ESI, q-TOF) *m/z* [M +H] calcd for $C_{15}H_{18}F_3NO_5S$ 382.0931; found 382.0931.

Methyl (*R*)-2-methyl-5-methylene-6,7,8,9-tetrahydro-5*H*-cyclohepta[*b*] pyridine-8-carboxylate (8)

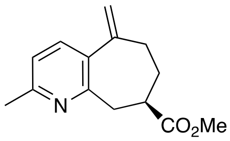

In a double-necked flask, K_2CO_3 (0.300 g, 2.17 mmol) and $Pd(PPh_3)_4$ (5 mol%, 0.026 g, 0.023 mmol) were added and put under Ar. CH_3CN (2 mL) was added and stirred. Triflate (**2**) (0.165 g, 0.433 mmol), was dissolved in CH_3CN (2.5 mL) and added to the solution. The reaction was refluxed for 6.5 hours when an additional 5 mol% catalyst was added (0.026 g $Pd(PPh_3)_4$, 0.023 mmol) and continued to reflux for additional 13 hours. The reaction was cooled and filtered over silica, washing with EtOAc (15 mL). The mixture was concentrated and crude product was purified using flash column chromatography (2:1 hexanes:EtOAc, R_f = 0.25). Bicyclic ester **8** was isolated as a clear, colorless oil (0.094 g, 0.41 mmol, 94%).

$[\alpha]^{20}_D$ -71.2 (*c* 0.94, $CHCl_3$)

Chiral GC: Method A: Retention times: 34.87 min. (major) and 35.04 min. (minor) 98% ee.

IR: 3073, 2943, 2855, 1730, 1590, 1435, 1165, 906, 828 cm^{-1}.

^{1}H NMR (500 MHz, $CDCl_3$): δ 7.40 (d, J = 7.8 Hz, 1H), 6.98 (d, J = 7.8 Hz, 1H), 5.18 (app s, 1H), 5.07 (app s, 1H), 3.69 (s, 3H), 3.26 (dd, J = 14.6, 1.4 Hz, 1H), 3.17 (dd, J = 14.7, 10.5 Hz, 1H), 2.76 (dddd, J = 9.8, 9.8, 4.3, 2.7 Hz, 1H), 2.65 (ddd, J = 14.0, 7.6, 3.8 Hz, 1H), 2.51 (s, 3H), 2.33 (ddd, J = 13.9, 9.8, 4.0 Hz, 1H), 2.15 (app dq, 1H), 2.00 (dddd, J = 13.6, 9.8, 9.8, 3.8 Hz, 1H) ppm.

^{13}C{^{1}H} NMR (125 MHz, $CDCl_3$): δ 175.7, 156.4, 156.0, 148.4, 136.0, 135.3, 121.3, 115.5, 51.8, 41.8, 40.5, 33.8, 32.9, 24.1 ppm

HRMS: (ESI, q-TOF) *m/z* [M +H] calcd for $C_{14}H_{17}NO_2$ 232.1332; found 232.1336.

Methyl (5*S*,8*R*)-2,5-dimethyl-6,7,8,9-tetrahydro-5*H*-cyclohepta[*b*]pyridine-8-carboxylate (9) and Methyl (5*R*,8*R*)-2,5-dimethyl-6,7,8,9-tetrahydro-5*H*-cyclohepta[*b*]pyridine-8-carboxylate (10)

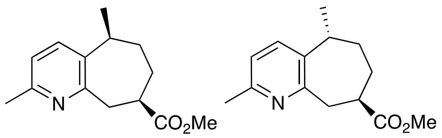

To a flask containing bicyclic pyridine **8** (0.080 g, 0.35 mmol), Wilkinson's catalyst (0.019 g, 0.021 mmol) was added, then the flask was put under Ar atmosphere. Dry CH_2Cl_2 (3 mL) was added, followed by NEt_3 (0.07 mL, 0.5 mmol). H_2 gas (12" balloon) was flushed through the flask, and a second balloon was used to maintain H_2 atmosphere overnight. After purging the flask with Ar, the mixture was filtered over silica using EtOAc to wash. Solvent was removed leaving a brown, clear oil. The crude mixture of the two desired diastereomers and undesired endocycle were purified using flash column chromatography (1:1 hexanes:EtOAc) to give a mixture of both diastereomers and trace endocycle.

Overlapping fractions were combined (0.048 g) and Pd/C (0.005 g, 10% w/w) was added and put under Ar atmosphere before addition of MeOH (6 mL). H_2 gas (12" balloon) was flushed through the flask, and a second balloon was used to maintain H_2 atmosphere overnight. The mixture was filtered over Celite and washed with EtOAc. The two products were purified using flash column chromatography (1:1 hexanes:EtOAc) and both **9** (0.040 g, 0.17 mmol) and **10** were isolated (0.030 g, 0.13 mmol) as clear, colorless oils (86% combined yield, 1.3: 1 dr of **9** and **10**)

Methyl (5*S*,8*R*)-2,5-dimethyl-6,7,8,9-tetrahydro-5*H*-cyclohepta[*b*]pyridine-8-carboxylate (9)

$[\alpha]^{20}_D$ -55.2 (*c* 0.27, $CHCl_3$)

Chiral GC Analysis: Method A: Retention times: 34.95 min. (major) and 35.12 min. (minor) 99% ee.

IR: 2933, 2353, 1732, 1590, 1462, 1161 cm^{-1}.

^{1}H NMR (500 MHz, $CDCl_3$): δ 7.30 (d, J = 7.8 Hz, 1H), 6.91 (d, J = 7.8 Hz, 1H), 3.61 (s, 3H), 3.36 (dd, J = 14.5, 9.7 Hz, 1H), 3.29 (dd, J = 14.7, 3.0 Hz, 1H), 2.99 (dddd, J = 14.3, 7.2, 7.2, 3.4 Hz, 1H), 2.65 (dddd, J = 9.6, 9.6, 3.2, 3.2 Hz, 1H), 2.48 (s, 3H) 2.12 (dddd, J = 14.0, 10.7, 9.4, 3.2 Hz, 1H) 1.98 (ddq, J = 14.0, 10.4, 3.5 Hz, 1H), 1.85 (dddd, J = 14.0, 6.9, 6.9, 3.2 Hz, 1H), 1.76 (dddd, J = 14.0, 10.7, 3.4, 3.4 Hz, 1H), 1.32 (d, J = 7.3 Hz, 3H) ppm.

^{13}C{^{1}H} NMR (125 MHz, $CDCl_3$): δ 175.9, 157.6, 154.9, 137.9, 136.2, 121.4, 51.4, 42.2, 40.8, 37.8, 32.4, 29.2, 24.0, 18.9 ppm.

HRMS (ESI, q-TOF) *m/z* [M +H] calcd for $C_{14}H_{19}NO_2$ 234.1489; found 234.1489.

Methyl (5*R*,8*R*)-2,5-dimethyl-6,7,8,9-tetrahydro-5*H*-cyclohepta[*b*]pyridine-8-carboxylate (10)

$[\alpha]^{20}_D$ -47.8 (*c* 0.09, $CHCl_3$)

Chiral GC: Method A: Retention times: 35.76 min. (minor) and 35.92 min. (major) 99% ee.

IR: 2930, 2851, 1734, 1464, 1433, 1159, 731 cm^{-1}.

^{1}H NMR (500 MHz, $CDCl_3$): δ 7.37 (d, J = 8.0 Hz, 1H), 6.98 (d, J = 7.8 Hz, 1H), 3.69 (s, 3H), 3.31 (dd, J = 14.0, 10.5 Hz, 1H), 3.25 (app d, 1H), 2.99 (app quin, 1H), 2.49 (s, 3H), 2.47 (dddd, J = 10.8, 10.8, 3.0, 3.0 1H), 2.18-2.12 (m, 1H), 1.97 (dddd, J = 11.8, 11.8, 11.8, 3.7 Hz, 1H), 1.88 (dddd, J = 14.0, 5.3, 3.8, 1.8 Hz, 1H), 1.34 (d, J = 7.0 Hz, 3H), 1.31-1.23 (m, 1H) ppm.

^{13}C NMR{^{1}H} (125 MHz, $CDCl_3$): δ 176.2, 158.9, 154.5, 137.8, 132.4, 121.2, 51.8, 42.1, 40.6, 35.0, 34.8, 33.9, 23.9, 20.4 ppm.

HRMS (ESI, q-TOF) *m/z* [M +H] calcd for $C_{14}H_{19}NO_2$ 234.1489; found 234.1488.

2-((5*R*,8*R*)-2,5-Dimethyl-6,7,8,9-tetrahydro-5*H*-cyclohepta[*b*]pyridin-8-yl)propan-2-ol [(+)-cananodine] (1)

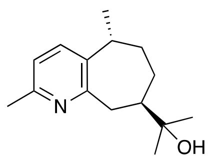

Ester **10** (0.057 g, 0.24 mmol) was put under Ar, then THF (5 mL) was added and cooled to -78 °C. MeLi (3.1 M in diethoxymethane, 0.80 mL, 2.60 mmol) was added dropwise and the mixture was stirred for 15 min. The reaction was removed from the dry ice bath and deemed complete by TLC (EtOAc) after an additional 15 min. Et_2O (20 mL) was added and the reaction quenched with sat. NH_4Cl. The organic layer was washed once with sat. NaCl, then dried over Na_2SO_4. Solvent was removed leaving product (**1**) as a clear, yellow gel. (0.056 g, 0.24 mmol, 99%).

$[\alpha]^{20}_D$ +12.0 (*c* 0.30, $CHCl_3$)

Chiral GC: Method A: Retention times: 36.54 min (minor) and 36.66 min (major). 99% ee.

IR: 3364, 2967, 2913, 2869, 1590, 1459, 1145, 920, 731 cm^{-1}.

^{1}H NMR (500 MHz, $CDCl_3$): δ 7.34 (d, J = 8.0 Hz, 1H), 6.93 (d, J = 8.0 Hz, 1H), 3.21 (app d, 1H), 2.97 (app quin 1H), 2.88 (dd, J = 13.4, 10.4 Hz, 1H), 2.48 (s, 3H), 2.14-2.08 (m, 1H), 1.89 (ddq, J = 13.7, 6.5, 4.9 Hz, 1H), 1.60 (dddd, J = 12.1, 12.1, 12.1, 3.6 Hz, 1H), 1.42 (dddd, J = 11.8, 10.3, 2.9, 1.5 Hz, 1H), 1.32 (d, J = 7.0 Hz, 3H), 1.26 (s, 3H), 1.25 (s, 3H), 1.30-1.21 (m, 1H) ppm.

^{13}C NMR (125 MHz, $CDCl_3$): δ 160.9, 154.3, 137.9, 132.4, 120.6, 73.3, 48.0, 39.8, 36.1, 35.3, 32.6, 27.6, 26.0, 23.9, 20.7 ppm.

HRMS (ESI, q-TOF) *m/z* [M +H] calcd for $C_{15}H_{23}NO$ 234.1852; found 234.1858.

(*R*)-3-((*S*)-2-((3-(Allyloxy)-6-methylpyridin-2-yl)methyl)hex-5-enoyl)-4-benzyloxazolidin-2-one (ent-3)

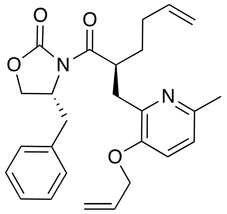

Oxazolidinone **ent-4** (0.743 g, 2.72 mmol) was put under Ar and THF (9 mL) was added and stirred. This solution was cooled to -78 °C, then LHMDS (2.75 mL, 1.0 M in THF, 2.73 mmol) was added dropwise then stirred for one hour. Next, picolyl bromide **5** (0.659 g, 2.72 mmol) was dissolved in THF (3 mL) and added immediately. This was stirred overnight, slowly warming to rt. The reaction was quenched with sat. NH_4Cl and the organic/aqueous layers were separated. The aqueous layer was extracted two times using CH_2Cl_2 and organic layers were combined and washed with sat. NaCl and dried over Na_2SO_4. The crude product was purified using flash column chromatography (9:1 hexanes:acetone) and pure product **ent-3** was isolated as a clear yellow oil. (0.812 g, 1.86 mmol, 69%).

$[\alpha]^{20}_D$ -35.4 (*c* 5.01, $CHCl_3$)

Chiral GC Analysis: Method A: Retention times: 40.675 min. (minor) and 42.162 min. (major) 96% de.

IR: 3083, 2918, 2853, 1780, 1692, 1455, 1386, 1347, 1258, 1191, 914, 730, 700 cm^{-1}.

^{1}H NMR (500 MHz, $CDCl_3$): δ 7.31 (app t, 2H), 7.25 (app t, 1H), 7.20 (app d, 2H), 6.96 (d, J = 8.2 Hz, 1H), 6.87 (d, J = 8.2 Hz, 1H), 6.06 (ddt, J = 17.2, 10.3, 5.0 Hz, 1H), 5.82 (dddd, J = 17.0, 10.2, 6.6, 6.6 Hz, 1H), 5.42 (dddd, J = 17.2, 1.7, 1.7, 1.7 Hz, 1H), 5.30 (dddd, J = 10.7,

1.7, 1.7, 1.7 Hz, 1H), 5.02 (dddd, J = 17.1, 1.7, 1.7, 1.7 Hz, 1H), 4.95 (app dd, 1H), 4.63 (dddd, J = 10.2, 6.6, 3.1, 3.1 Hz, 1H), 4.53 (ddd, J = 5.0, 1.7, 1.7 Hz, 2H), 4.50 (dddd, J = 11.0, 6.8, 6.8, 4.3 Hz, 1H), 4.14-4.08 (m, 2H), 3.35 (dd, J = 13.3, 3.2 Hz, 1H), 3.31 (dd, J = 15.7, 10.1 Hz, 1H), 3.16 (dd, J = 15.6, 4.3 Hz, 1H), 2.53 (dd, J = 13.3, 10.5 Hz, 1H), 2.38 (s, 3H), 2.25-2.13 (m, 2H), 1.88 (dddd, J = 13.3, 10.0, 6.6, 6.6 Hz, 1H), 1.72 (dddd, J = 13.3, 9.4, 6.5, 6.5 Hz, 1H) ppm.

^{13}C{^{1}H} NMR (125 MHz, $CDCl_3$): δ 176.7, 153.0, 150.6, 148.4, 148.2, 138.2, 136.1, 133.1, 129.4, 128.9, 127.1, 121.0, 118.4, 117.5, 114.8, 69.0, 65.8, 55.9, 40.2, 37.9, 34.3, 32.0, 31.4, 23.5 ppm.

HRMS (ESI, q-TOF) *m/z* [M +H] calcd for $C_{26}H_{30}N_2O_4$ 435.2278; found 435.2281.

Methyl (*S*)-2-((3-(allyloxy)-6-methylpyridin-2-yl)methyl)hex-5-enoate (ent-7)

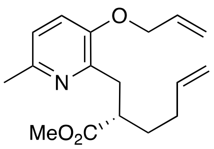

LiOH•H_2O (0.144 g, 3.42 mmol) was placed in a flask and H_2O (2 mL) was added. This was cooled to 0 °C and H_2O_2 (0.67 mL (30%), 8.6 mmol) was added and stirred for 30 minutes. Oxazolidinone **ent-3** (0.741 g, 1.71 mmol) was dissolved in THF (8 mL, final ratio 4:1, THF: H_2O) then added to the mixture. The reaction was stirred overnight and deemed complete by TLC. $Na_2S_2O_3$ was used to quench the excess peroxides, stirring at 0 °C for 15 minutes. The solution was acidified to pH = 6 with NH_4Cl and acetic acid and the crude product was extracted from aqueous layer with EtOAc four times. The combined organic layers were washed with sat. NaCl and dried over Na_2SO_4. Solvent was removed using rotary evaporation, and the crude acid product was dissolved in MeOH (15 mL) and 8 drops of conc. H_2SO_4 was added. The reaction was stirred and refluxed overnight. After 24 hours, the reaction was complete by TLC. Solvent was removed and pH was neutralized using $NaHCO_3$ after dilution in EtOAc which was used to extract product three times from the aqueous layer. The combined organic layers were washed once with sat. NaCl and then dried over Na_2SO_4. After solvent was removed, the crude ester was purified using flash column chromatography using 3:1 hexanes:EtOAc. Pure ester (**ent-7**) was isolated as a clear, yellow oil (0.364 g, 1.26 mmol, 74%).

$[\alpha]^{20}_{D}$ +9.3 (*c* 5.02, $CHCl_3$)

Chiral GC Analysis: Method A: Retention times: 37.60 min. (major) and 37.62 min. (minor) 99% ee.

IR: 3082, 2945, 2863, 1733, 1458, 1255, 1161, 995, 916, 814 cm^{-1}.

^{1}H NMR (500 MHz, $CDCl_3$): δ 6.97 (d, J = 8.3 Hz, 1H), 6.89 (d, J = 8.4 Hz, 1H), 6.03 (ddt, J = 17.2, 10.2, 5.0 Hz, 1H), 5.77 (dddd, J = 17.0, 10.3, 6.8, 6.8 Hz, 1H), 5.41 (dddd, J = 17.3, 1.7, 1.7, 1.7 Hz, 1H), 5.29 (dddd, J = 10.6, 1.6, 1.6, 1.6 Hz, 1H), 4.99 (dddd, J = 17.3, 1.9, 1.9, 1.9

Hz, 1H), 4.93 (dddd. J = 10.2, 1.2, 1.2, 1.2 Hz, 1H), 4.51 (ddd, J = 5.1, 1.7, 1.7 Hz, 2H), 3.64 (s, 3H), 3.13 (dd, J = 14.2, 8.3 Hz, 1H), 3.03 (dd, J = 14.2, 6.2 Hz, 1H), 2.98 (dddd, J = 8.6, 8.6, 6.3, 6.3 Hz, 1H), 2.42 (s, 3H), 2.15-2.01 (m, 2H), 1.80 (dddd, J = 14.6, 8.8, 8.8, 5.8 Hz, 1H), 1.62 (dddd, J = 11.4, 9.6, 6.4, 4.9 Hz, 1H) ppm.

$^{13}C\{^1H\}$ NMR (125 MHz, $CDCl_3$): δ 176.4, 150.5, 148.9, 148.4, 138.1, 133.0, 121.1, 118.8, 117.5, 114.8, 68.9, 51.3, 43.6, 34.6, 31.5, 31.2, 23.4 ppm.

HRMS (ESI, q-TOF) *m/z* [M +H] calcd for $C_{17}H_{23}NO_3$ 290.1751; found 290.1750.

Methyl (*S*)-2-((6-methyl-3-(((trifluoromethyl)sulfonyl)oxy)pyridin-2-yl)methyl)hex-5-enoate (ent-2)

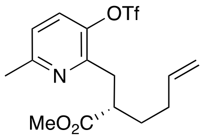

$Pd(PPh_3)_4$ (0.015 g, 0.013 mmol) was washed with MeOH and dried using vacuum filtration before addition to a flask containing ester (**ent-3**) (0.364 g, 1.26 mmol) and K_2CO_3 (0.522 g, 3.78 mmol). After establishing Ar atmosphere, MeOH (15 mL) was added and stirred overnight at room temperature. After this time, the mixture was filtered over Celite washing with EtOAc, followed by solvent removal. After diluting in CH_2Cl_2, NH_4Cl was added to neutralize pH. Product was extracted from the aqueous layer three times with CH_2Cl_2, then the combined organic layers were washed with sat. NaCl. Solvent was removed to give the crude phenol as a hazy orange oil (0.343g) which was added to a flask containing $PhNTf_2$ (0.630 g, 1.76 mmol) and put under Ar. Dry CH_2Cl_2 (12 mL) was added and stirred before NEt_3 (0.28 mL, 2.02 mmol) was added. This mixture was left to stir at room temperature overnight. Once deemed complete by TLC, the dark, cloudy blue solution was washed with 10% NaOH, sat. NH_4Cl, and sat. NaCl sequentially. This product was dried over Na_2SO_4, and solvent was removed to give a thick blue oil. The crude product was purified by flash column chromatography using 6:1 hexanes:EtOAc, leaving **ent-2** as a clear, colorless oil (0.428 g, 1.12 mmol, 93%).

$[\alpha]^{20}_D$ -1.4 (*c* 4.12, $CHCl_3$)

Chiral GC Analysis: Method A: Retention times: 34.15 min. (minor) and 34.21 min. (major) 98% ee.

IR: 3081, 2952, 2854, 1735, 1458, 1425, 1210, 1136, 862 cm^{-1}

^1H NMR (500 MHz, $CDCl_3$): δ 7.43 (d, J = 8.4 Hz, 1H), 7.05 (d, J = 8.5 Hz, 1H), 5.78 (dddd, J = 17.0, 10.3, 6.6, 6.6 Hz, 1H), 5.03 (app dd, 1H), 4.97 (app d, 1H), 3.66 (s, 3H), 3.22 (dd, J = 15.0, 8.9 Hz, 1H), 3.09-3.04 (m, 1H), 3.00 (dd, J = 15.0, 5.2 Hz, 1H), 2.52 (s, 3H), 2.17-2.06 (m, 2H), 1.76 (app sextet, 1H), 1.69-1.62 (m, 1H) ppm.

$^{13}C\{^1H\}$ NMR (125 MHz, $CDCl_3$): δ 175.7, 157.9, 151.2, 143.1, 137.6, 128.9, 122.3, 118.5 (q, J = 320.2 Hz), 115.3, 51.6, 43.0, 33.7, 31.4, 31.3, 24.0 ppm.

HRMS (ESI, q-TOF) *m/z* [M +H] calcd for $C_{15}H_{18}F_3NO_5S$ 382.0931; found 382.0932.

Methyl (*S*)-2-methyl-5-methylene-6,7,8,9-tetrahydro-5*H*-cyclohepta[*b*]pyridine-8-carboxylate (ent-8)

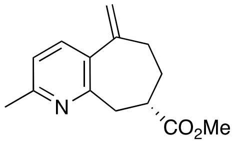

$Pd(PPh_3)_4$ was washed with MeOH, then dried using vacuum filtration to give shiny yellow crystals (0.068 g, 0.060 mmol) which was added to a culture tube containing K_2CO_3 (0.822 g, 5.94 mmol) and put under Ar. CH_3CN (5 mL) was added and stirred. Triflate **ent-2** (0.452 g, 1.19 mmol) was dissolved in CH_3CN (4 mL) and added to the tube. The reaction was refluxed for 5 hours when an additional 5 mol% catalyst was added (0.068 g $Pd(PPh_3)_4$, 0.060 mmol) and continued to reflux for additional 16 hours. At this time, the reaction mixture was cooled and filtered over silica, washing with EtOAc (100 mL). The mixture was concentrated and the crude brown oil was purified using flash column chromatography (2:1 hexanes:EtOAc, R_f = 0.25). Pure bicyclic pyridine **ent-8** was isolated as a clear, colorless oil (0.191 g, 0.819 mmol, 69%).

$[\alpha]^{20}_D$ +70.4 (*c* 0.76, $CHCl_3$)

Chiral GC Analysis: Method A: Retention times: 34.87 min. (minor) and 35.04 min. (major) 99% ee.

IR: 3082, 2950, 1863, 1733, 1590, 1169, 906, 733 cm^{-1}.

^1H NMR (500 MHz, $CDCl_3$): δ 7.40 (d, J = 7.8 Hz, 1H), 6.98 (d, J = 7.8 Hz, 1H), 5.18 (app s, 1H), 5.06 (d, J = 1.7 Hz, 1H), 3.69 (s, 3H), 3.26 (dd, J = 14.7, 1.4 Hz, 1H), 3.17 (dd, J = 14.5, 10.5 Hz, 1H), 2.76 (dddd, J = 10.1, 10.1, 4.3, 2.6 Hz, 1H), 2.66 (ddd, J = 14.0, 7.7, 3.9 Hz, 1H), 2.51 (s, 3H), 2.33 (ddd, J = 13.8, 9.6, 3.9 Hz, 1H), 2.15 (app dq, 1H), 2.00 (dddd, J = 13.6, 9.8, 9.8, 3.8 Hz, 1H) ppm.

^{13}C NMR$\{^1H\}$ (125 MHz, $CDCl_3$): δ 175.6, 156.4, 156.0, 148.4, 136.0, 135.2, 121.3, 115.5, 51.7, 41.8, 40.5, 33.8, 32.9, 24.1 ppm.

HRMS (ESI, q-TOF) *m/z* [M +H] calcd for $C_{14}H_{17}NO_2$ 232.1332; found 232.1333.

Methyl (5*R*,8*S*)-2,5-dimethyl-6,7,8,9-tetrahydro-5*H*-cyclohepta[*b*]pyridine-8-carboxylate (ent-9) and **Methyl (5*S*,8*S*)-2,5-dimethyl-6,7,8,9-tetrahydro-5*H*-cyclohepta[*b*]pyridine-8-carboxylate (ent-10)**

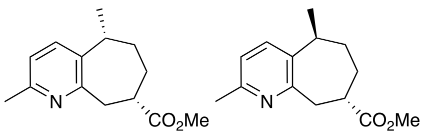

To a flask containing starting alkene **ent-8** (0.231 g, 1.00 mmol), Wilkinson's catalyst (0.046 g, 0.050 mmol) was added, then put under Ar atmosphere. Dry CH_2Cl_2 (10 mL) was added, followed by NEt_3 (0.21 mL, 1.50 mmol). H_2 gas (12" balloon) was flushed through the flask, and a second balloon was used to maintain H_2 atmosphere overnight. After purging the flask with Ar, the mixture was filtered over silica using EtOAc. The solvent was removed leaving a brown, clear oil. The crude mixture of the two desired diastereomers and undesired endocycle were purified using flash column chromatography (1:1 hexanes:EtOAc) to give a mixture of both diastereomers and trace endocycle. Overlapping fractions were combined (0.207 g) and Pd/C (0.023 g, 10% w/w) was added and put under Ar atmosphere before addition of MeOH (12 mL). H_2 gas (12" balloon) was flushed through the flask, and a second balloon was used to maintain H_2 atmosphere overnight. The mixture was filtered over Celite and washed with EtOAc. The two products were purified using flash column chromatography (1:1 hexanes: EtOAc) and both the (*5S,8S*) diastereomer **ent-9** (0.085 g, 0.36 mmol) and the (*5R,8S*) diastereomer **ent-10** (0.117 g, 0.50 mmol) were isolated as clear, colorless oils (87% combined yield, 1.4:1 dr)

Methyl (5*R*,8*S*)-2,5-dimethyl-6,7,8,9-tetrahydro-5*H*-cyclohepta[*b*]pyridine-8-carboxylate (ent-9)

$[\alpha]^{20}_D$ +53.2 (*c* 1.16, $CHCl_3$)

Chiral GC Analysis: Method A: Retention times: 34.97 min. (minor) and 35.14 min. (major). 99% ee.

IR: 2921.9, 2847.3, 1733.8, 1460.4, 1436.5, 1157.8, 729.6 cm^{-1}.

^{1}H NMR (500 MHz, $CDCl_3$): δ 7.30 (d, J = 7.8 Hz, 1H), 6.92 (d, J = 7.8 Hz, 1H), 3.65 (s, 3H), 3.36 (dd, J = 14.7, 9.8 Hz, 1H), 3.29 (dd, J = 14.6, 3.0 Hz, 1H), 2.99 (dddd, J = 14.4, 7.2, 7.2, 3.4 Hz, 1H), 2.64 (dddd, J = 9.4, 9.4, 3.2, 3.2 Hz, 1H), 2.48 (s, 3H), 2.12 (dddd, J = 14.0, 10.7, 9.3, 3.2 Hz, 1H), 1.99 (ddq, J = 14.1, 10.6, 3.6 Hz, 1H), 1.84 (dddd, J = 14.0, 6.8, 6.8, 3.2 Hz, 1H), 1.76 (dddd, J = 14.1, 10.7, 3.4, 3.4 Hz, 1H), 1.32 (d, J = 7.4 Hz, 3H) ppm.

^{13}C{^{1}H} NMR (125 MHz, $CDCl_3$): δ 175.7, 157.4, 154.8, 137.7, 136.1, 121.2, 51.7, 42.0, 40.6, 37.6, 32.2, 29.1, 23.9, 18.8 ppm.

HRMS (ESI, q-TOF) *m/z* [M +H] calcd for $C_{14}H_{19}NO_2$ 234.1489; found 234.1486..

Methyl (5*S*,8*S*)-2,5-dimethyl-6,7,8,9-tetrahydro-5*H*-cyclohepta[*b*]pyridine-8-carboxylate (ent-10)

$[\alpha]^{20}_D$ +53.0 (*c* 0.10, $CHCl_3$)

Chiral GC Analysis: Method A: Retention times: 35.76 min. (major) and 35.92 min. (minor) 99% ee.

IR: 3059.6, 2919.7, 2848.7, 1730.4, 1464.1, 1433.0, 1159.1, 729.5 cm^{-1}

^{1}H NMR (500 MHz, $CDCl_3$): δ 7.37 (d, J = 8.0 Hz, 1H), 6.98 (d, J = 7.8 Hz, 1H), 3.69 (s, 3H), 3.31 (dd, J = 14.0, 10.5 Hz, 1H), 3.25 (app d, 1H), 2.99 (app quin, 1H), 2.49 (s, 3H), 2.47 (dddd, J = 10.8, 10.8, 3.1, 3.1 Hz, 1H), 2.18-2.12 (m, 1H) 1.97 (dddd, J = 11.7, 11.7, 11.7, 4.2 Hz, 1H), 1.88 (dddd, J = 13.9, 5.3, 3.7, 1.7 Hz, 1H), 1.34 (d, J = 7.0 Hz, 3H), 1.31-1.23 (m, 1H) ppm.

^{13}C{^{1}H} NMR (125 MHz, $CDCl_3$): δ 176.1, 158.8, 154.5, 137.7, 132.4, 121.2, 51.7, 42.1, 40.5, 35.0, 34.8, 33.9, 23.9, 20.4 ppm.

HRMS (ESI, q-TOF) *m/z* [M +H] calcd for $C_{14}H_{19}NO_2$ 234.1489; found 234.1488.

2-((5*S*,8*S*)-2,5-Dimethyl-6,7,8,9-tetrahydro-5*H*-cyclohepta[*b*]pyridin-8-yl)propan-2-ol [(-)-cananodine] (ent-1)

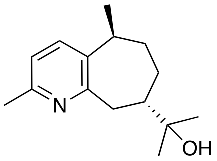

Ester **ent-10** (0.075 g, 0.32 mmol) was put under Ar, then THF (12 mL) was added and the solution was cooled to -78 °C. MeLi (3.1 M in diethoxymethane, 1.10 mL, 3.55 mmol) was added dropwise and stirred for 1 hour. TLC (EtOAc) indicated all starting material was consumed. The reaction was diluted in Et_2O (20 mL), then quenched with sat. NH_4Cl. H_2O (5 mL) was added to dissolve resultant white precipitate. The layers were separated and the organic fraction was washed once with sat. NaCl, then dried over Na_2SO_4. Solvent removal by rotary evaporation gave pure product **ent-1** as a clear, yellow gel. (0.070 g, 0.30 mmol, 93%).

$[\alpha]^{20}_D$ -11.6 (*c* 0.44, $CHCl_3$)

Chiral GC Analysis: Method A: Retention times: 36.54 min (major) and 36.64 min (minor). 99% ee.

IR: 3366, 2965, 2917, 2870, 1642, 1458, 1380, 1175, 922, 723 cm^{-1}.

^{1}H NMR (500 MHz, $CDCl_3$): δ 7.34 (d, J = 7.7 Hz, 1H), 6.93 (d, J = 7.8 Hz, 1H), 3.22 (app d, 1H), 2.97 (app quin 1H), 2.88 (dd, J = 13.2, 10.2 Hz, 1H), 2.48 (s, 3H), 2.12-2.08 (m, 1H), 1.89 (ddq, J = 13.7, 6.4, 5.0 Hz, 1H), 1.60 (dddd, J = 12.1, 12.1, 12.1, 3.6 Hz, 1H), 1.44-1.39 (m, 1H), 1.32 (d, J = 7.0 Hz, 3H), 1.26 (s, 3H), 1.25 (s, 3H), 1.29-1.21 (m, 1H) ppm.

$^{13}C\{^1H\}$ NMR (125 MHz, $CDCl_3$): *δ* 160.9, 154.2, 137.9, 132.4, 120.7, 73.3, 48.0, 39.7, 36.1, 35.3, 32.6, 27.6, 26.0, 23.9, 20.7 ppm.

HRMS (ESI, q-TOF) *m/z* [M +H] calcd for $C_{15}H_{23}NO$ 234.1852; found 234.1857.

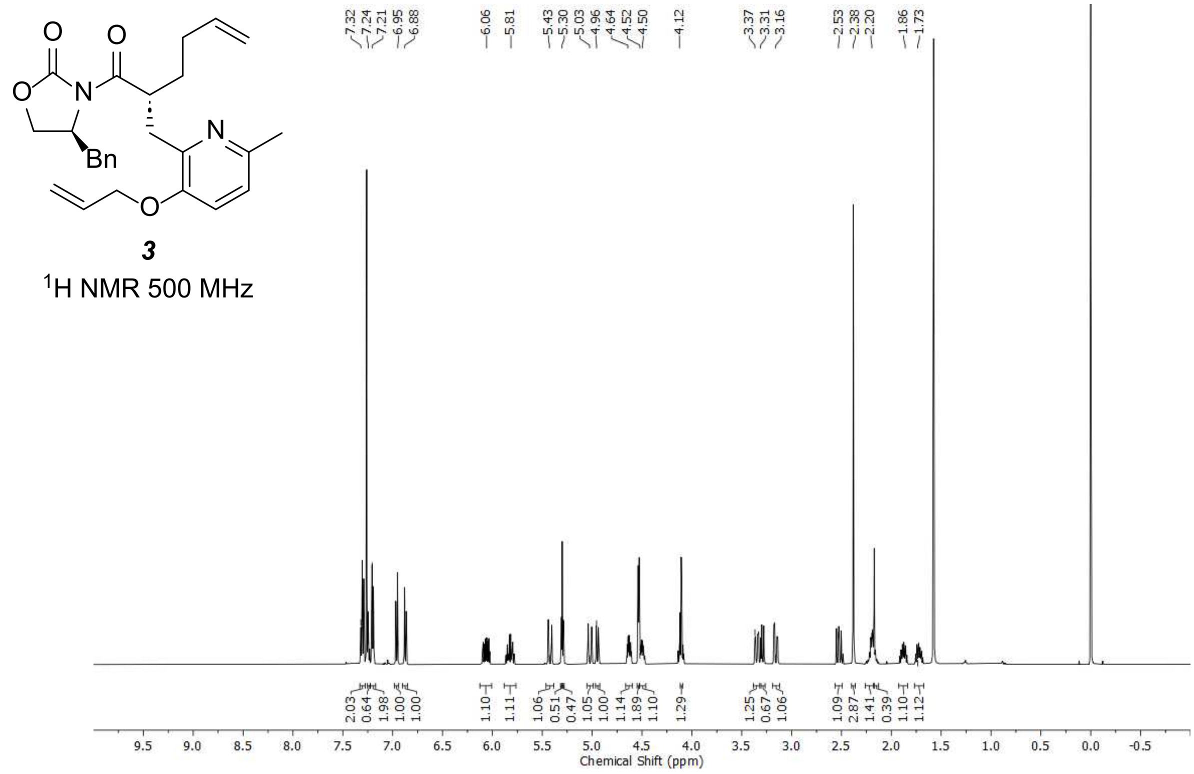

Figure S1. ^{1}H NMR Spectrum of ***3***

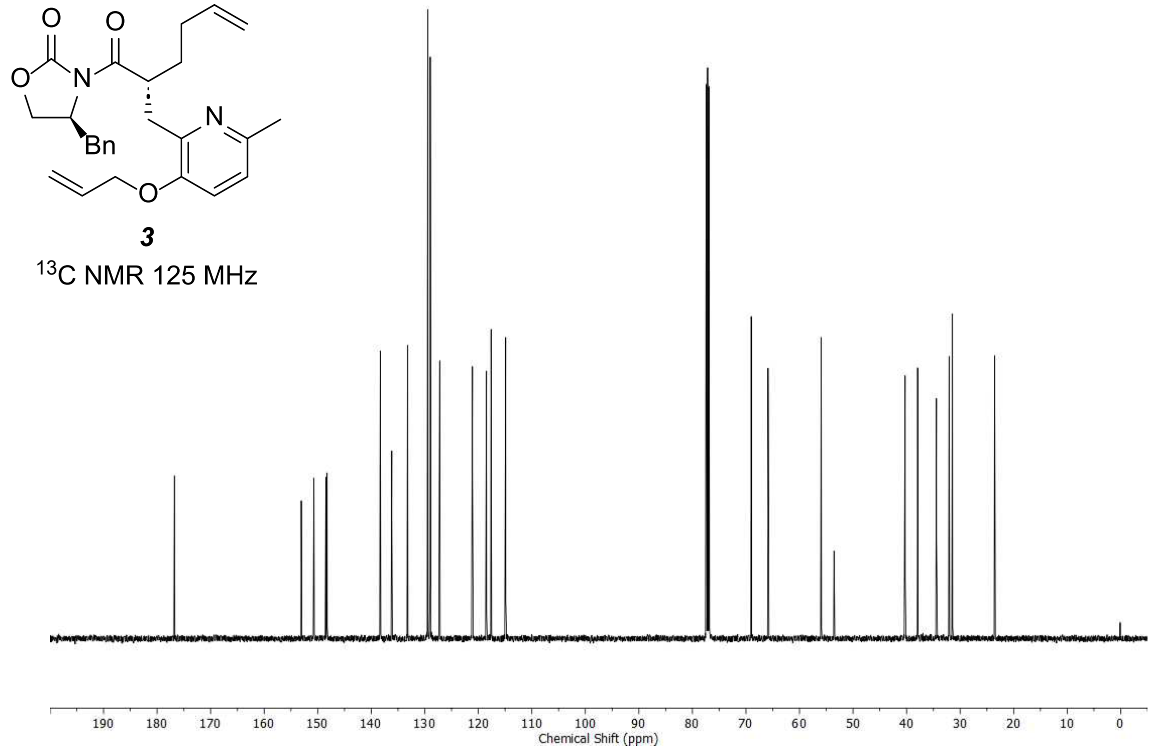

Figure S2. ^{13}C NMR Spectrum of ***3***

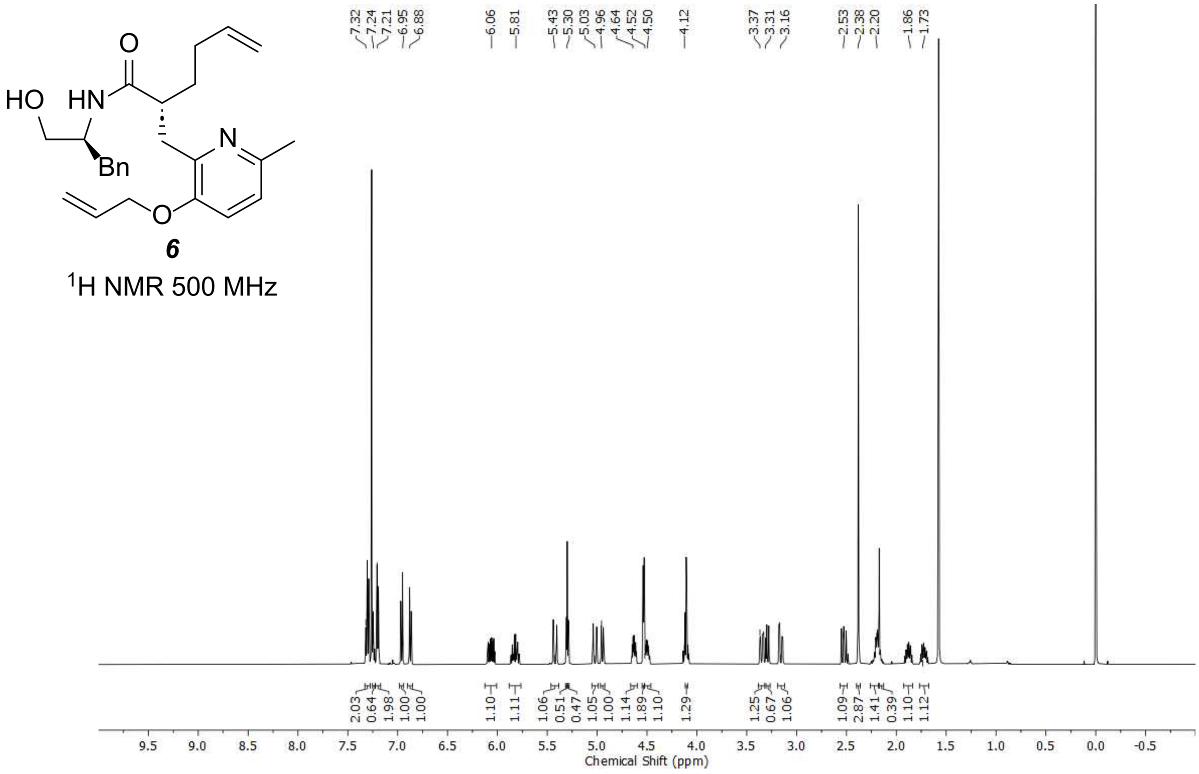

Figure S3. ^{1}H NMR Spectrum of ***6***

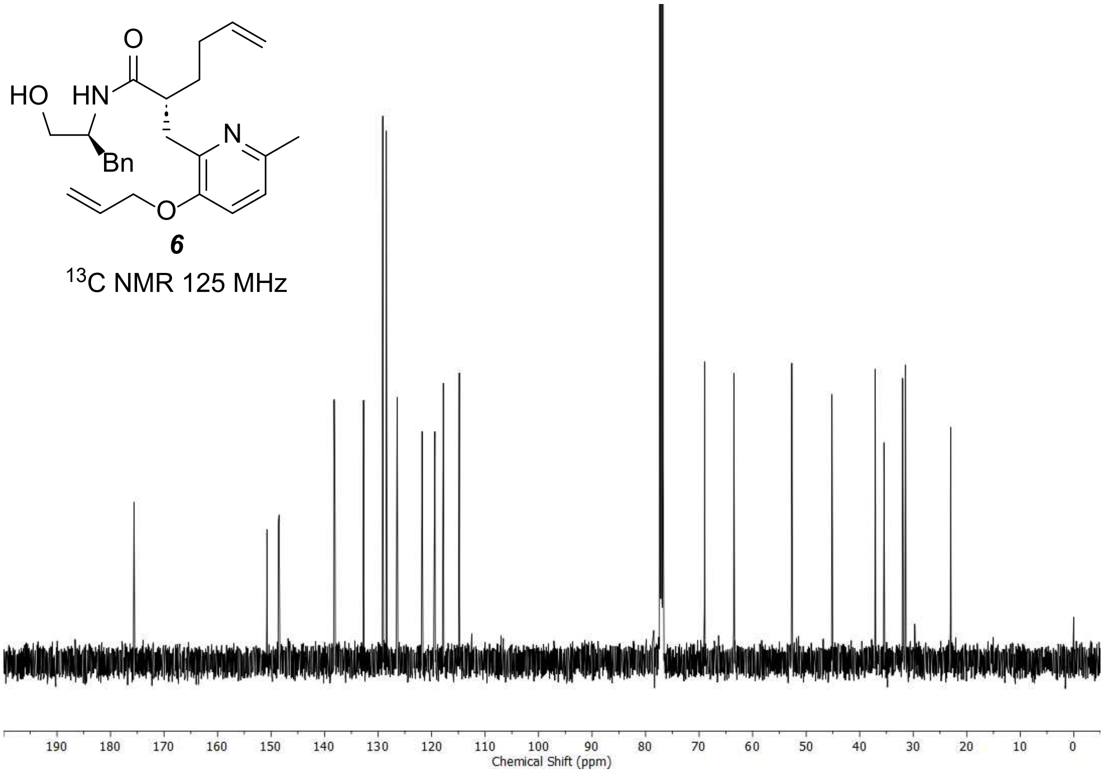

Figure S4. ^{13}C NMR Spectrum of ***6***

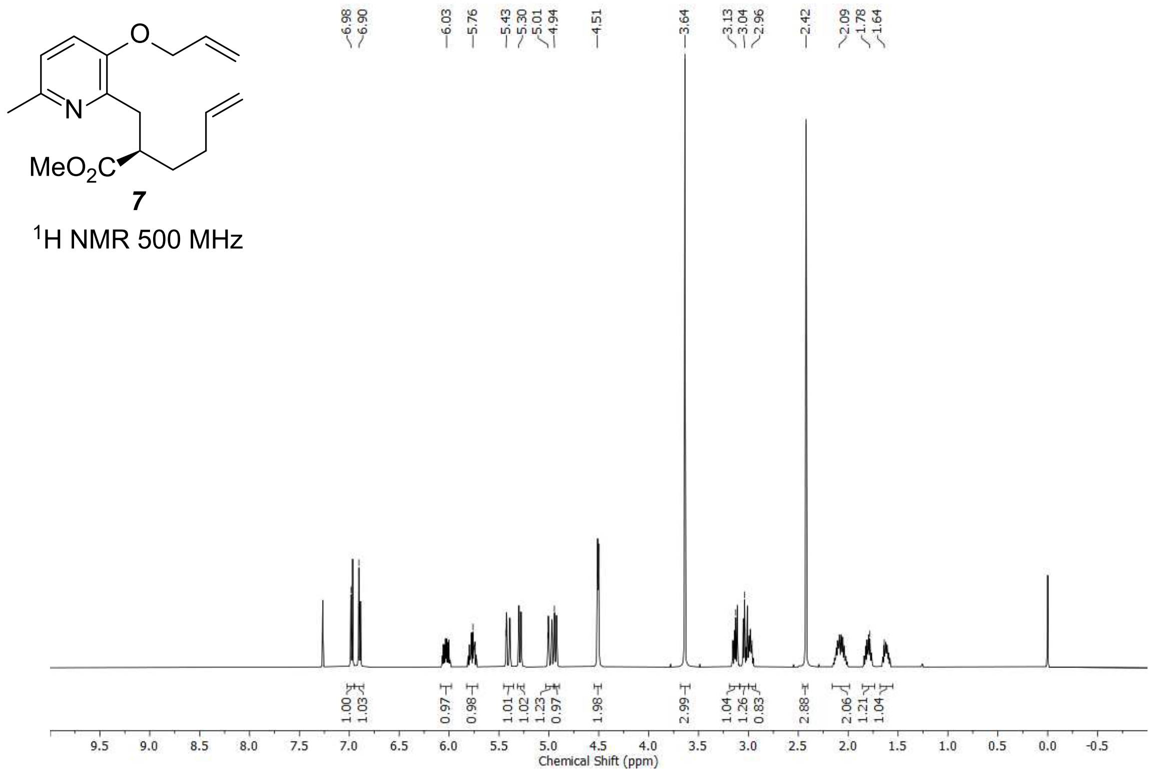

Figure S5. ^{1}H NMR Spectrum of *7*

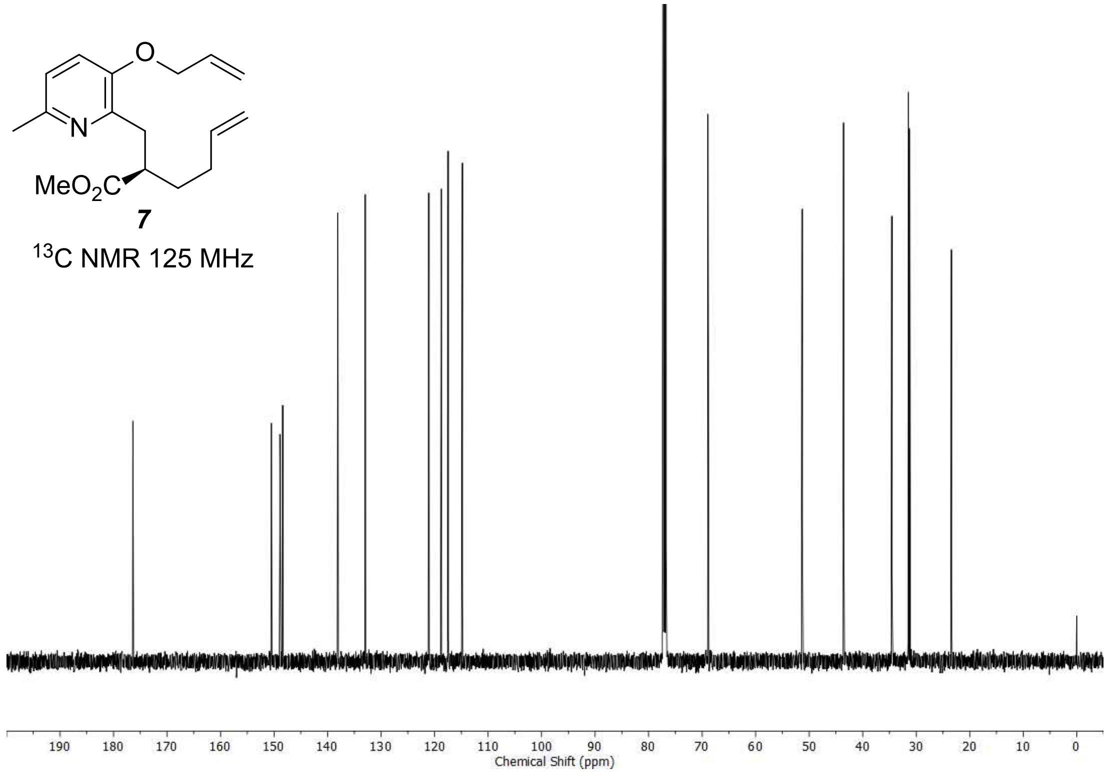

Figure S6. ^{13}C NMR Spectrum of *7*

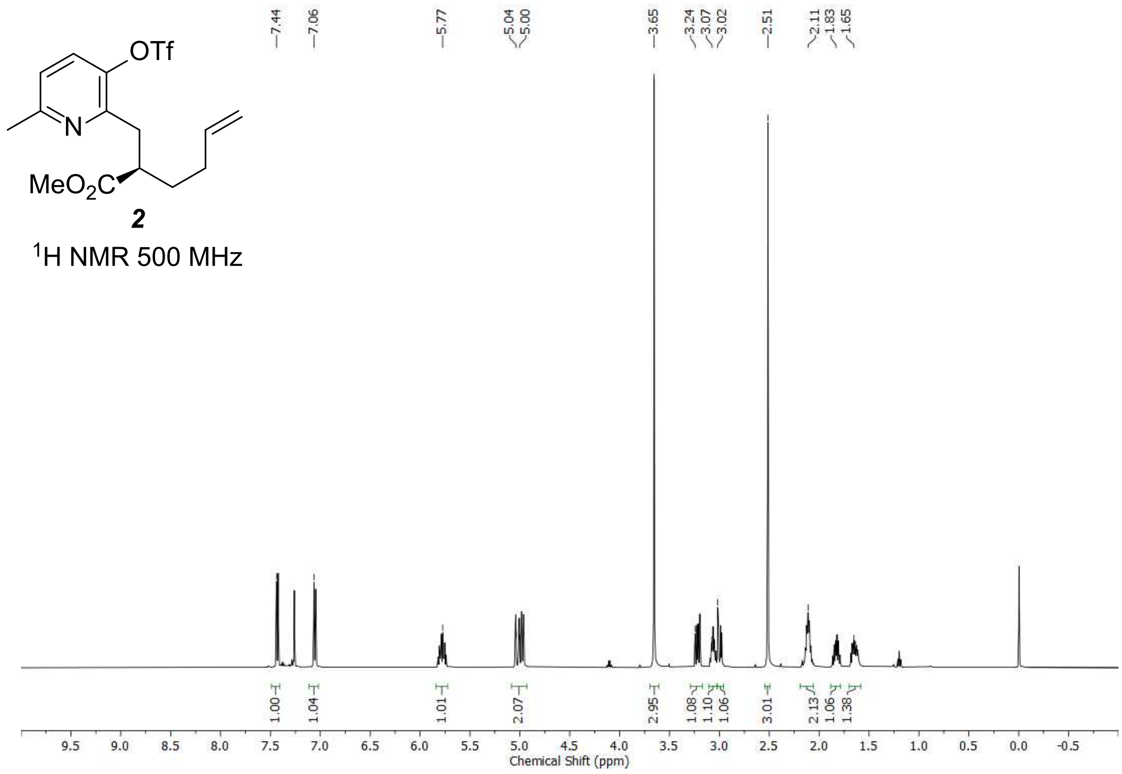

Figure S7. ^{1}H NMR Spectrum of ***2***

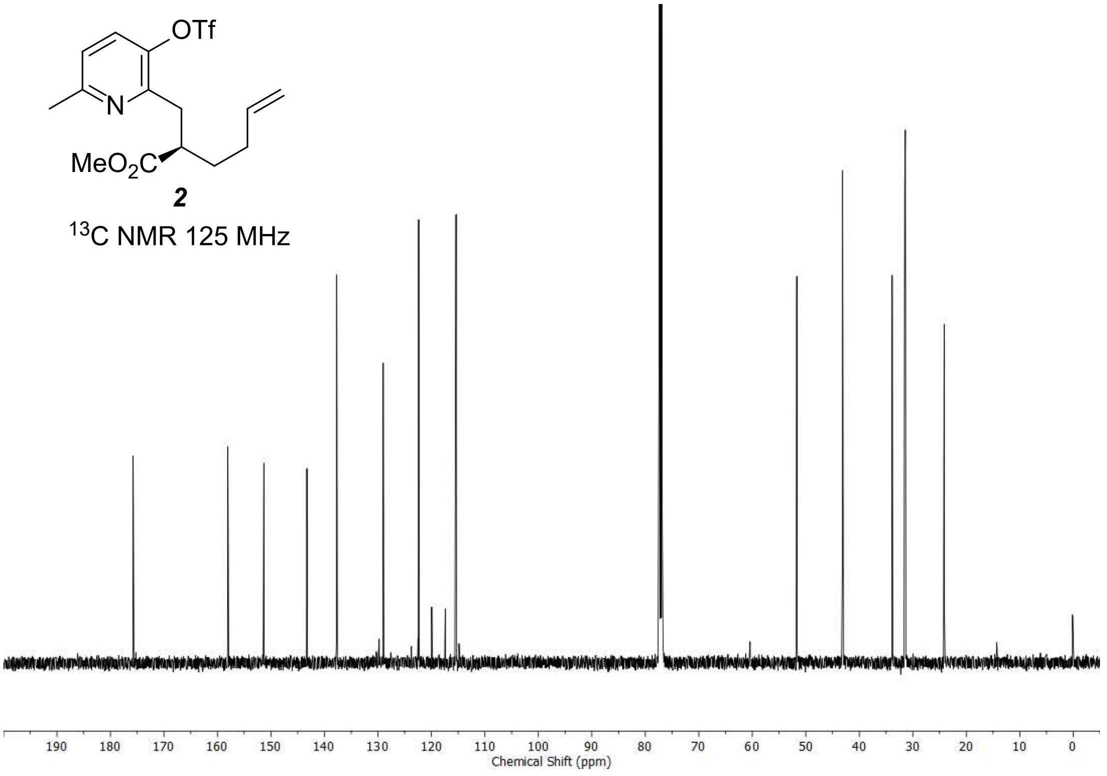

Figure S8. ^{13}C NMR Spectrum of ***2***

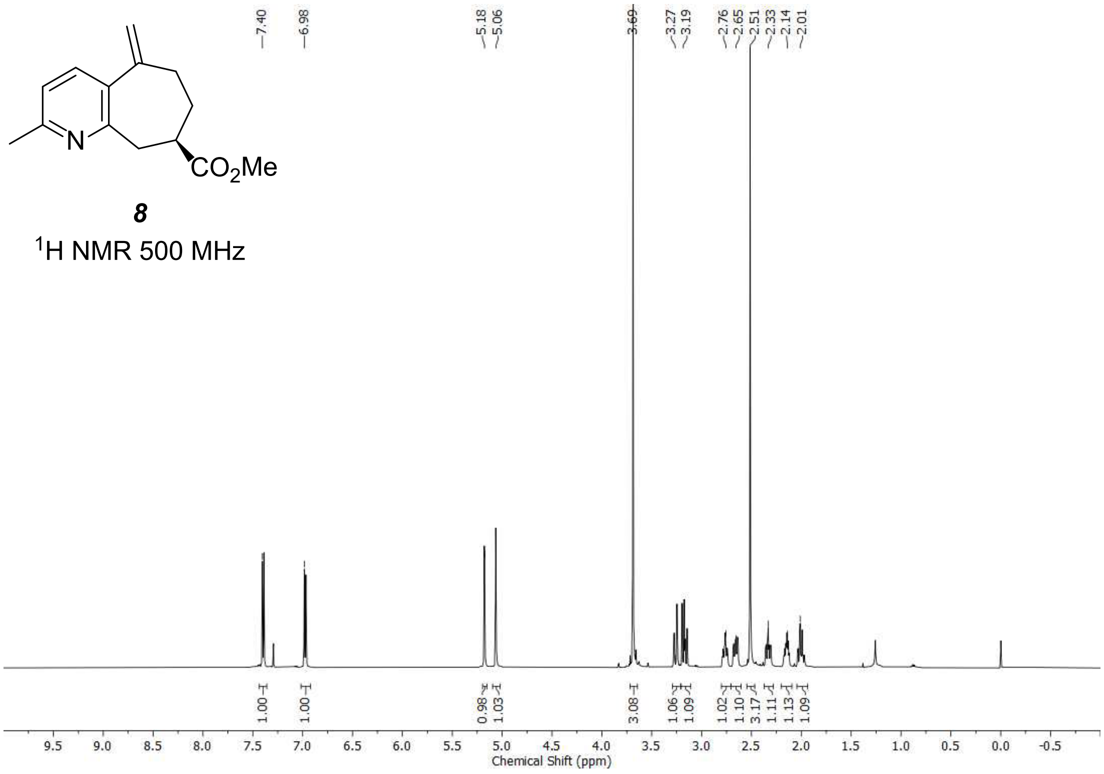

Figure S9. ^{1}H NMR Spectrum of ***8***

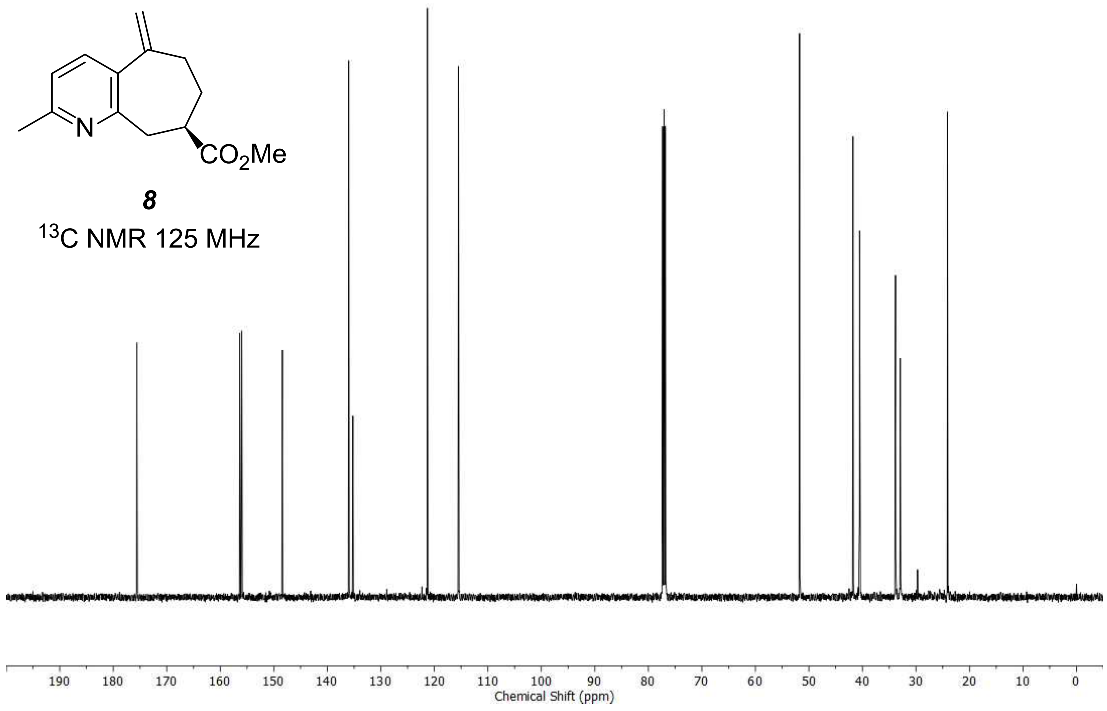

Figure S10. ^{13}C NMR Spectrum of ***8***

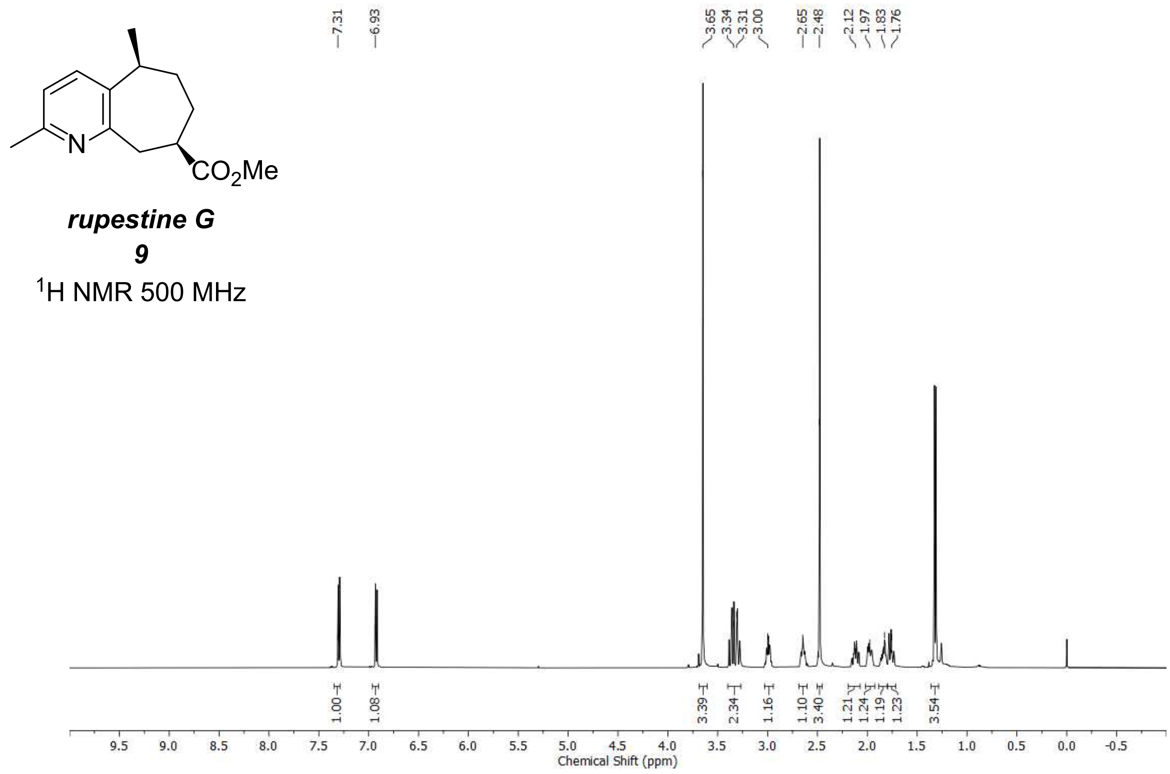

Figure S11. ^{1}H NMR Spectrum of ***9***

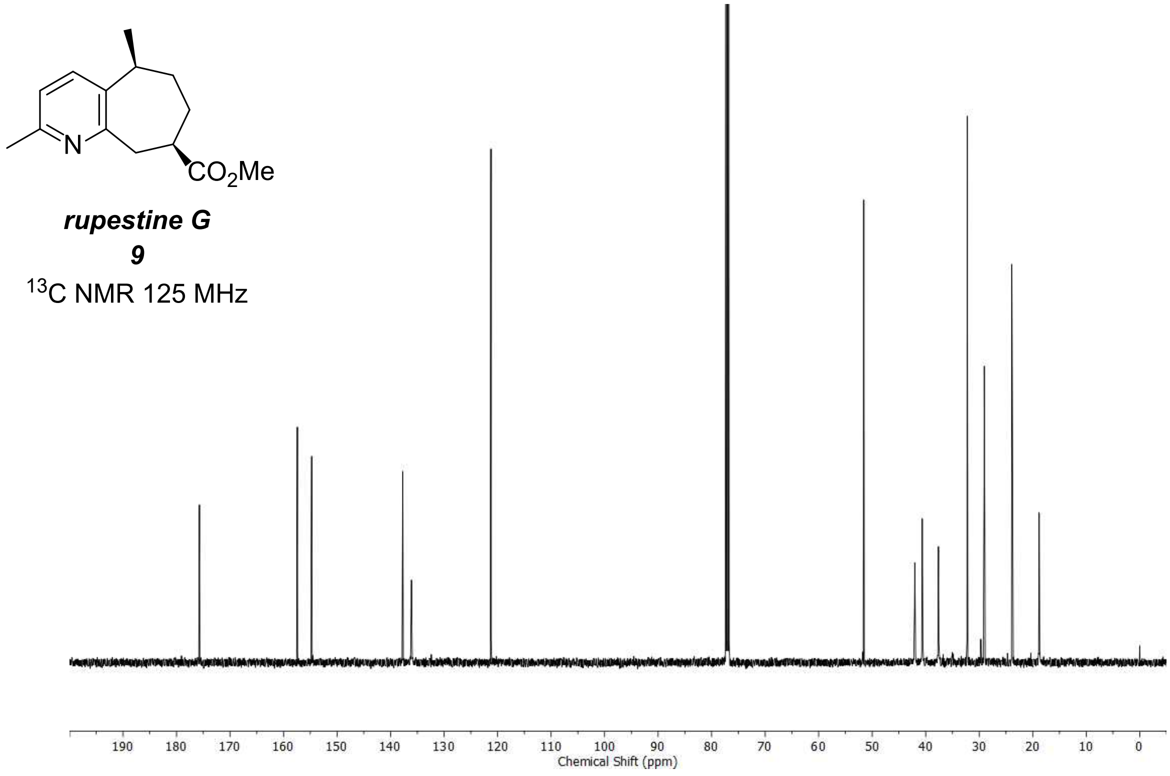

Figure S12. ^{13}C NMR Spectrum of ***9***

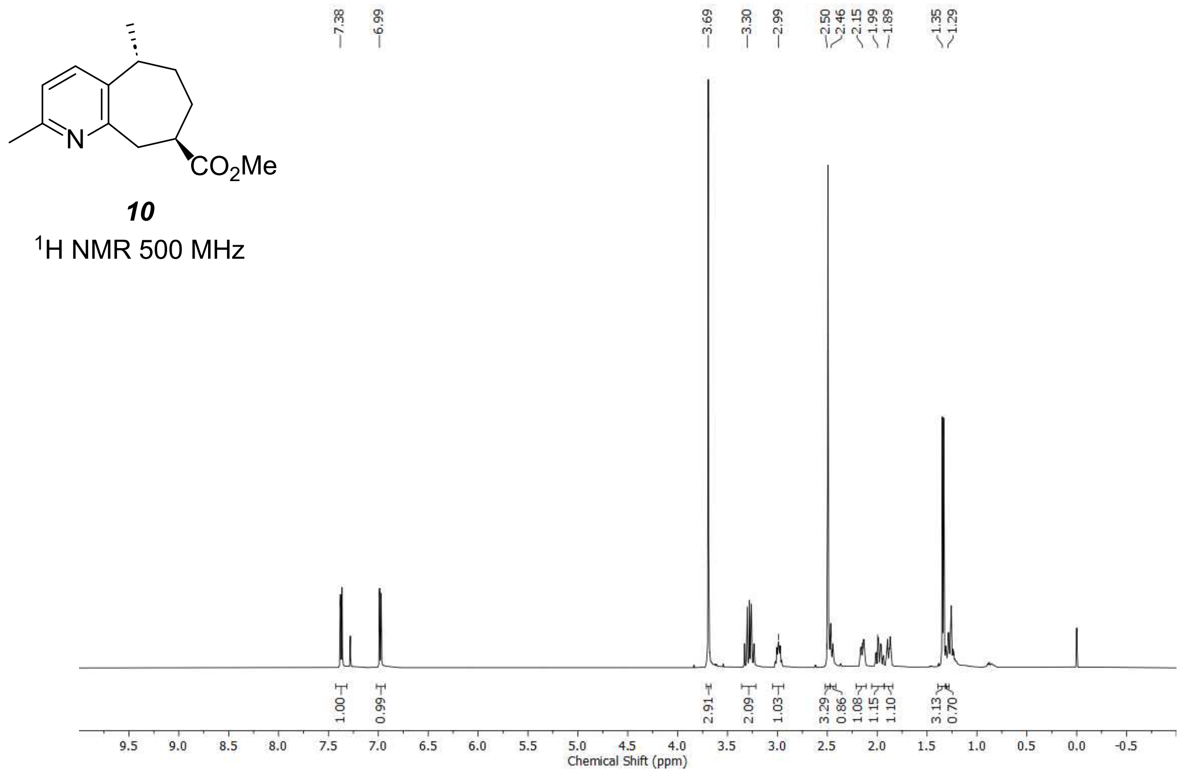

Figure S13. ^{1}H NMR Spectrum of ***10***

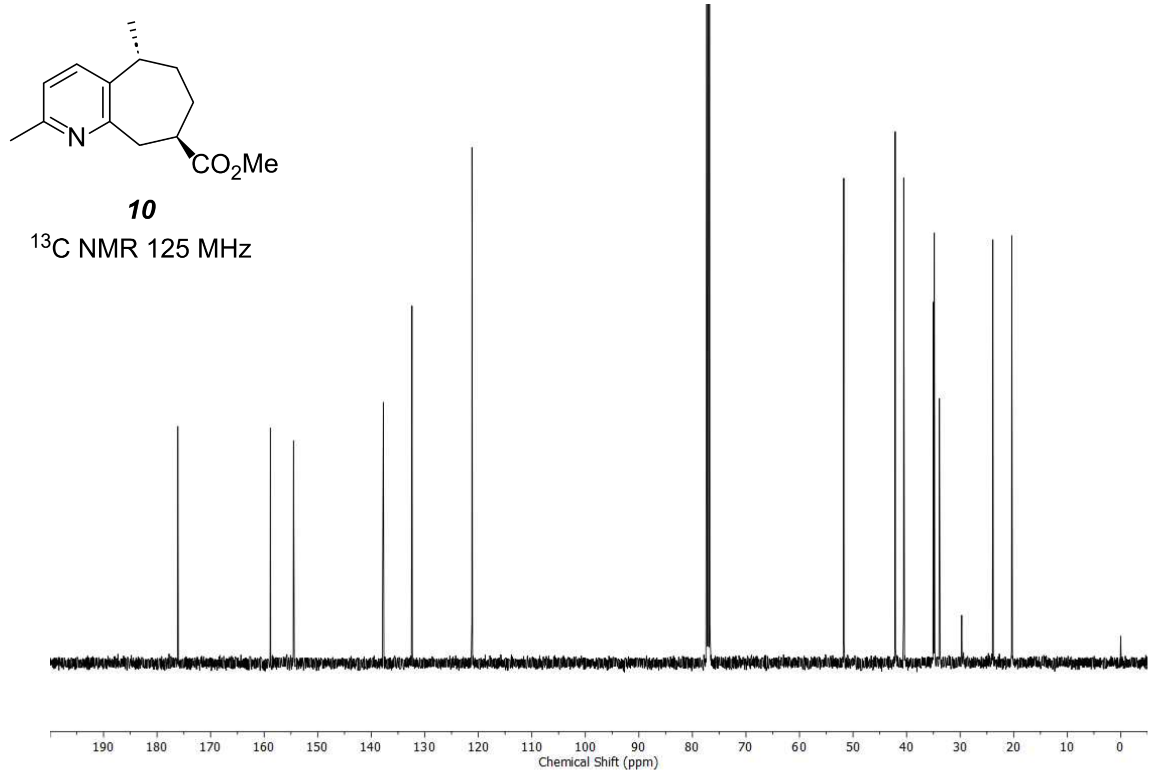

Figure S14. ^{13}C NMR Spectrum of ***10***

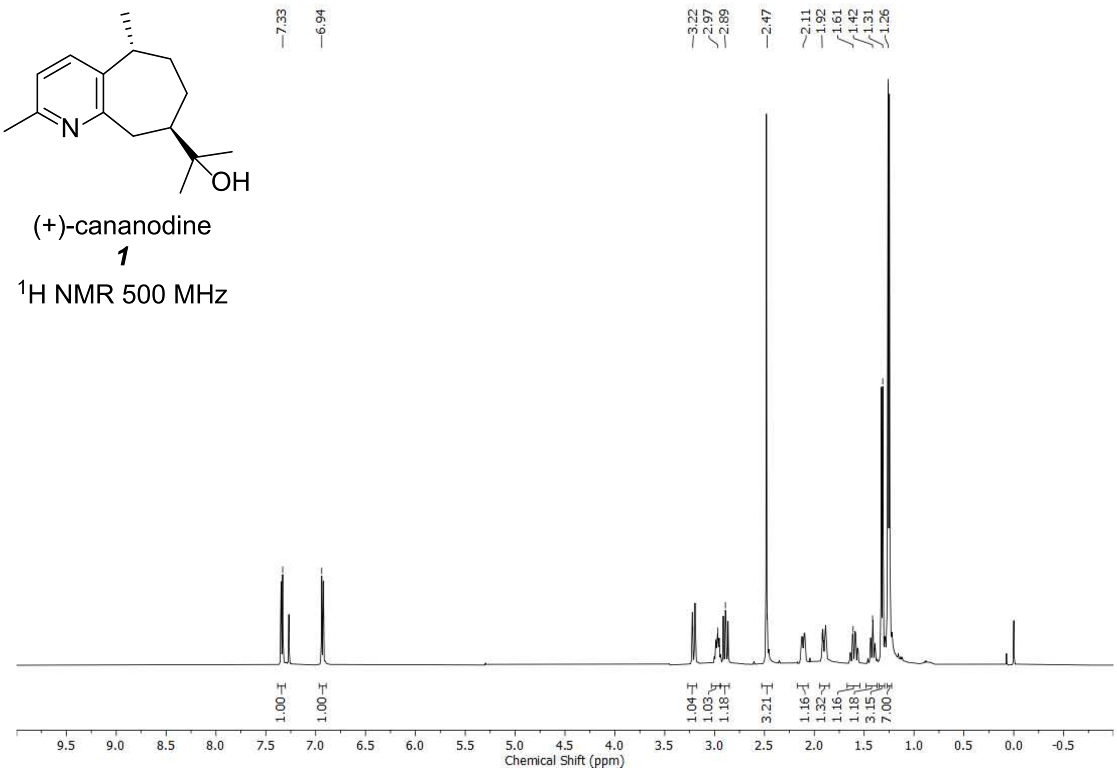

Figure S15. ^{1}H NMR Spectrum of (+)-Cananodine (***1***)

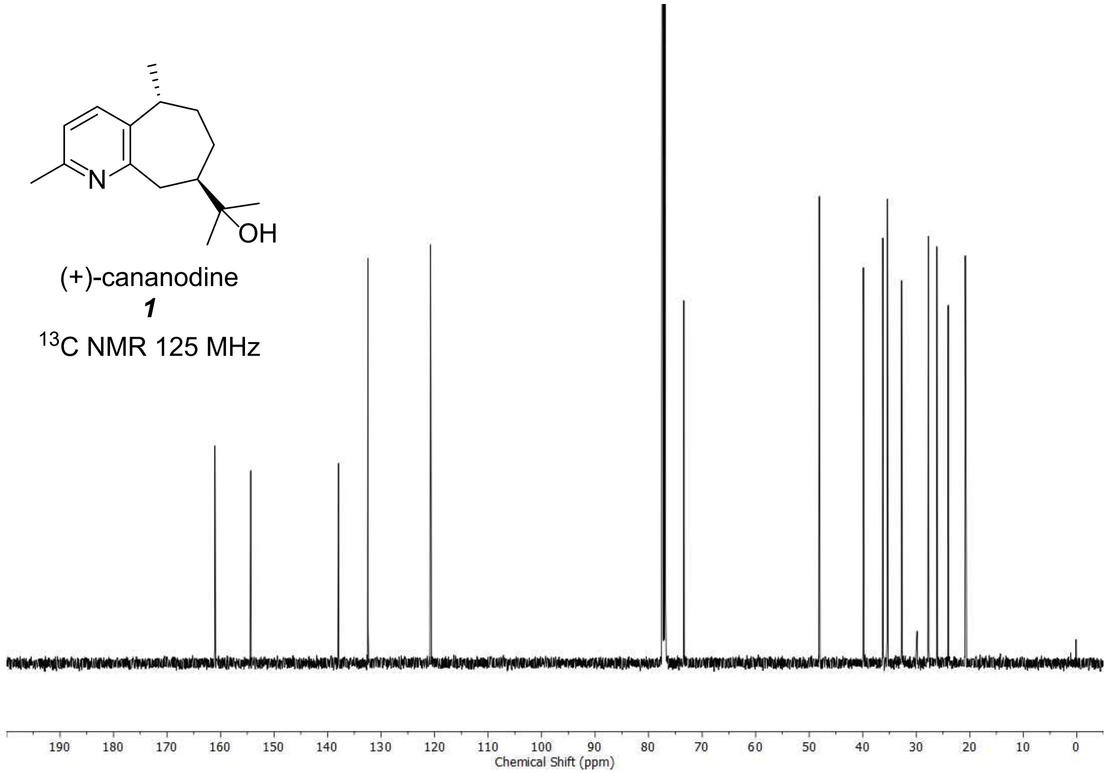

Figure S16. ^{13}C NMR Spectrum of (+)-Cananodine (***1***)

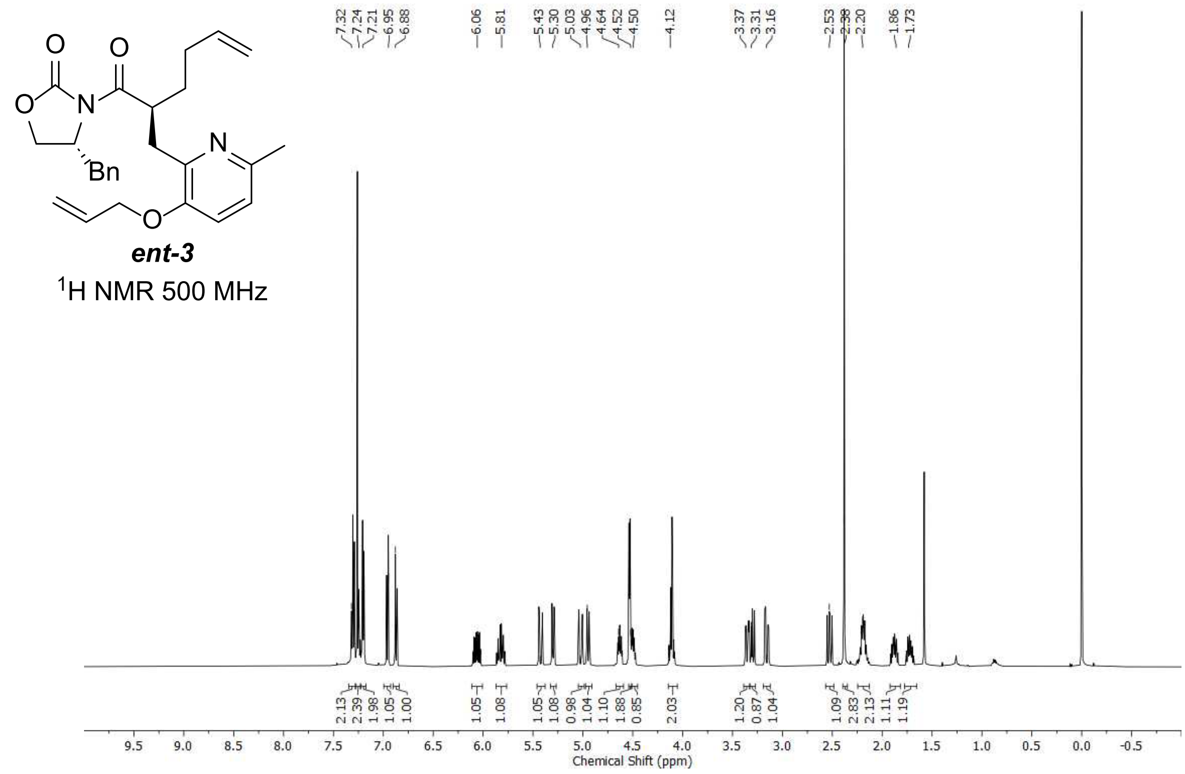

Figure S17. ^{1}H NMR Spectrum of ***ent-3***

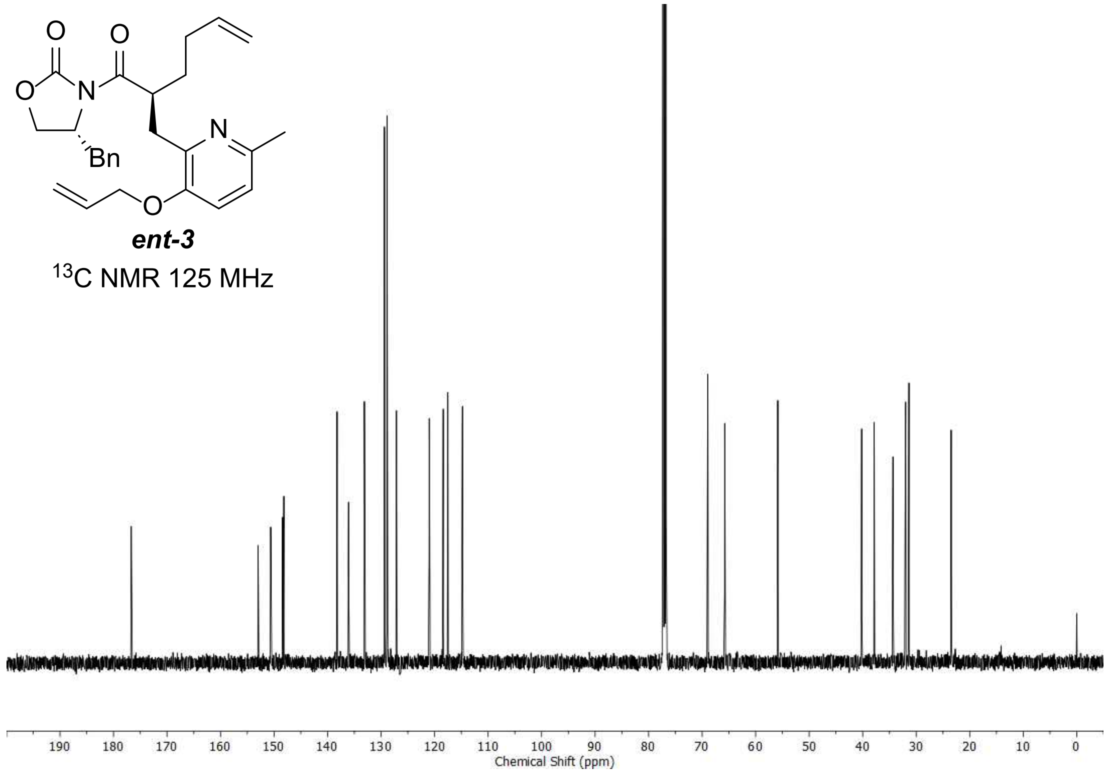

Figure S18. ^{13}C NMR Spectrum of ***ent-3***

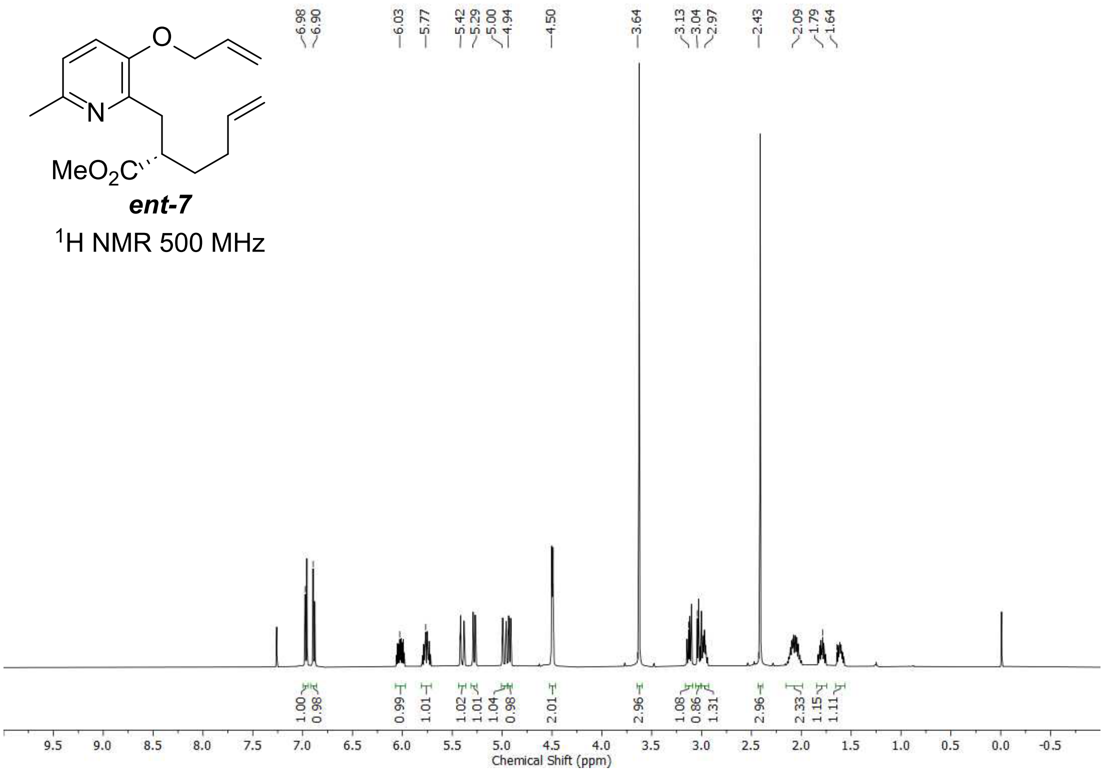

Figure S19. ^{1}H NMR Spectrum of ***ent-7***

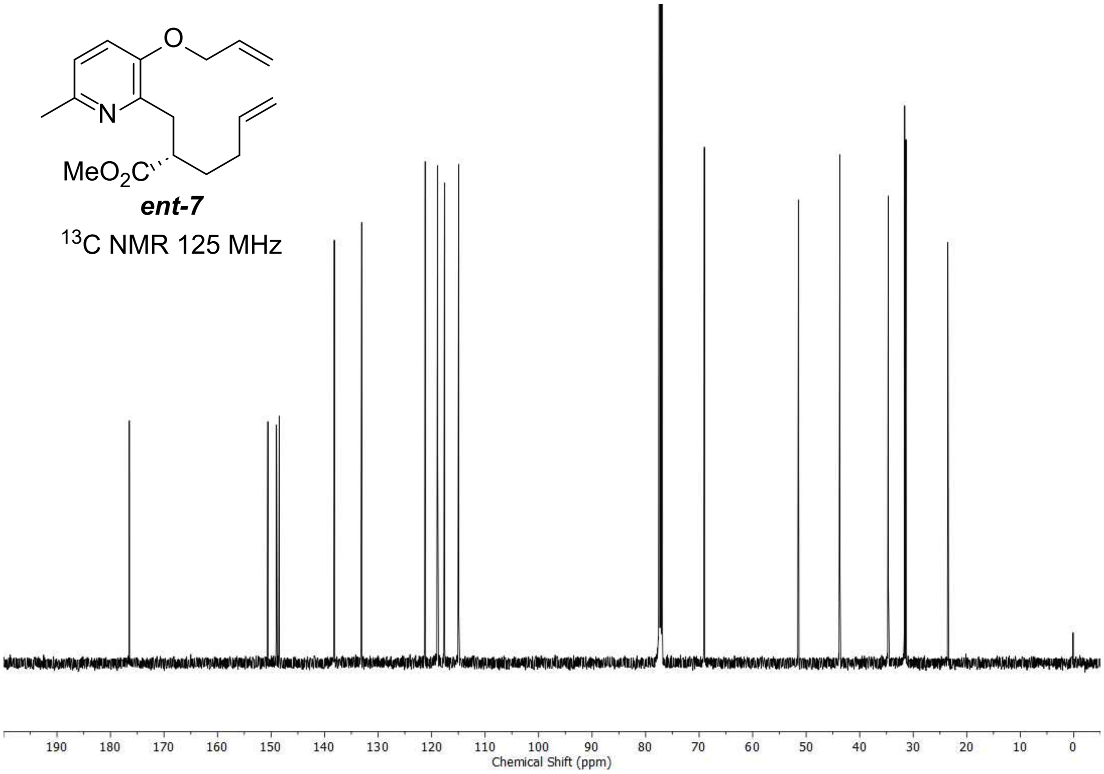

Figure S20. ^{13}C NMR Spectrum of ***ent-7***

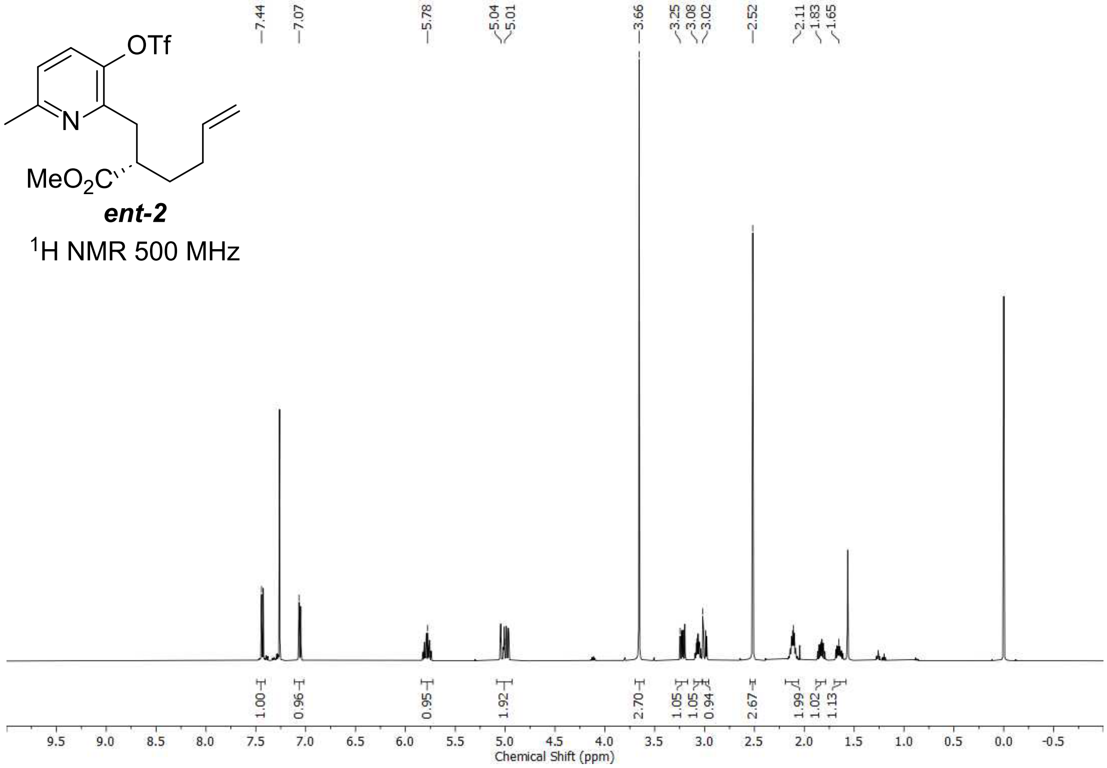

Figure S21. ^{1}H NMR Spectrum of ***ent-2***

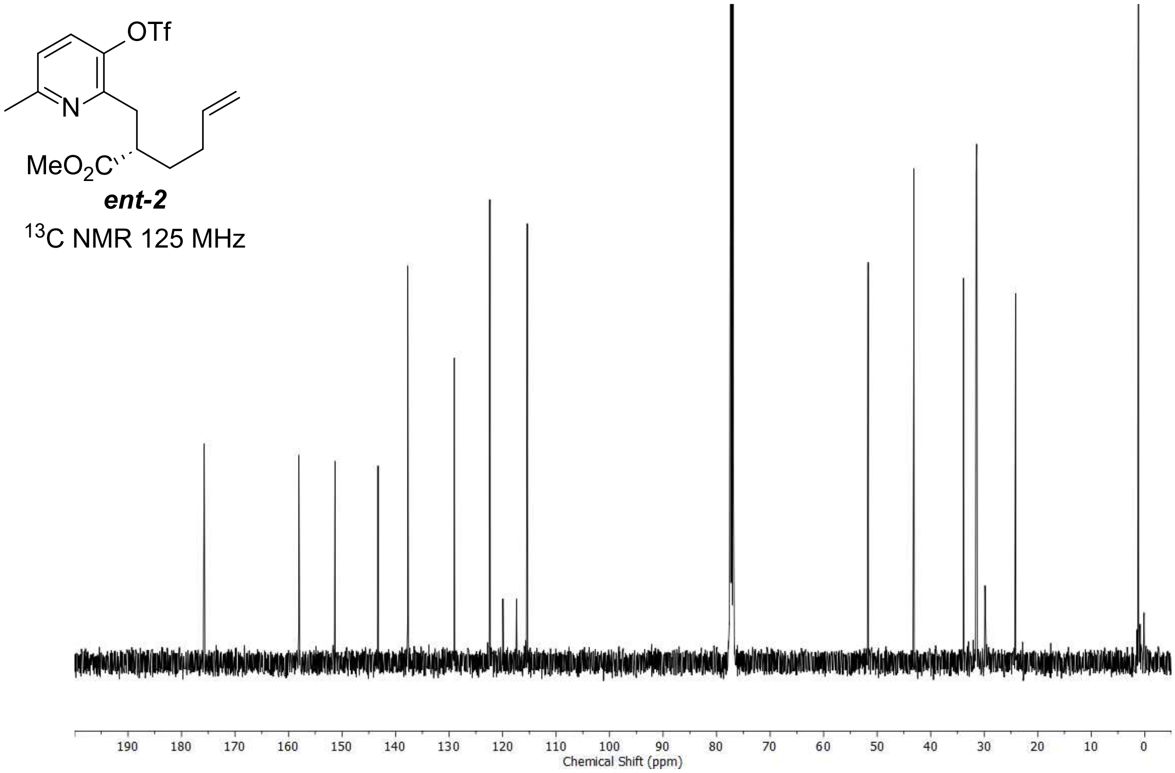

Figure S22. ^{13}C NMR Spectrum of ***ent-2***

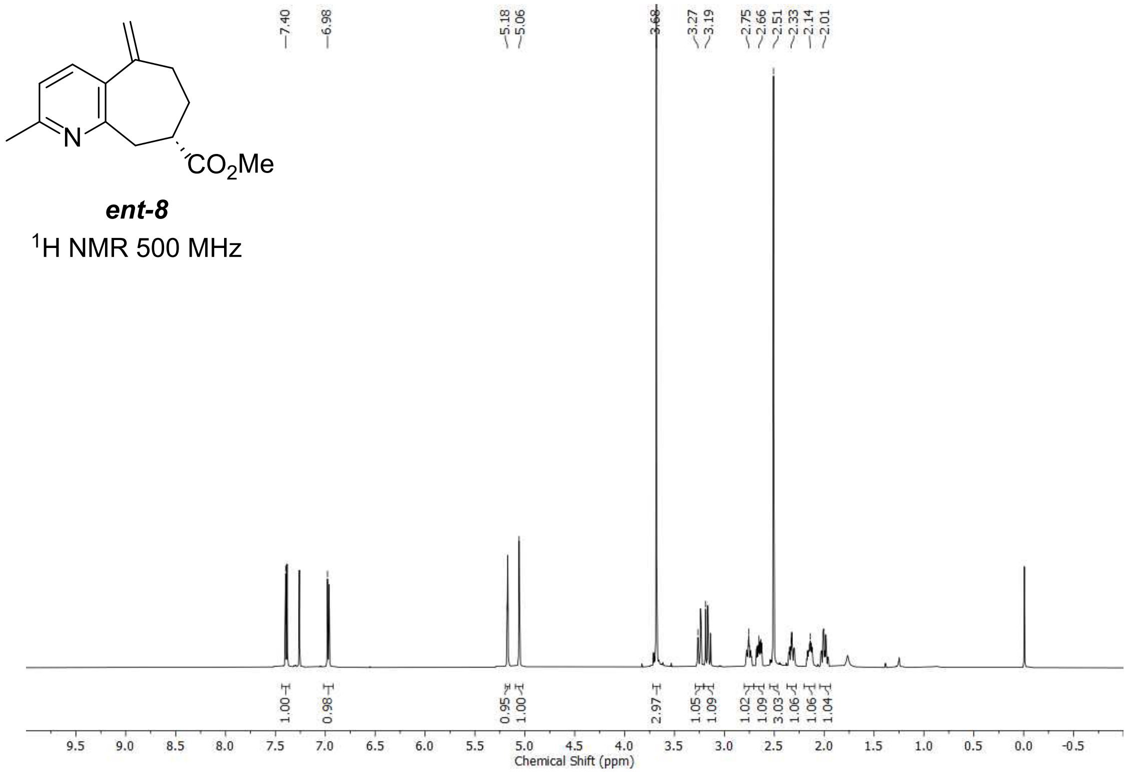

Figure S23. ^{1}H NMR Spectrum of ***ent-8***

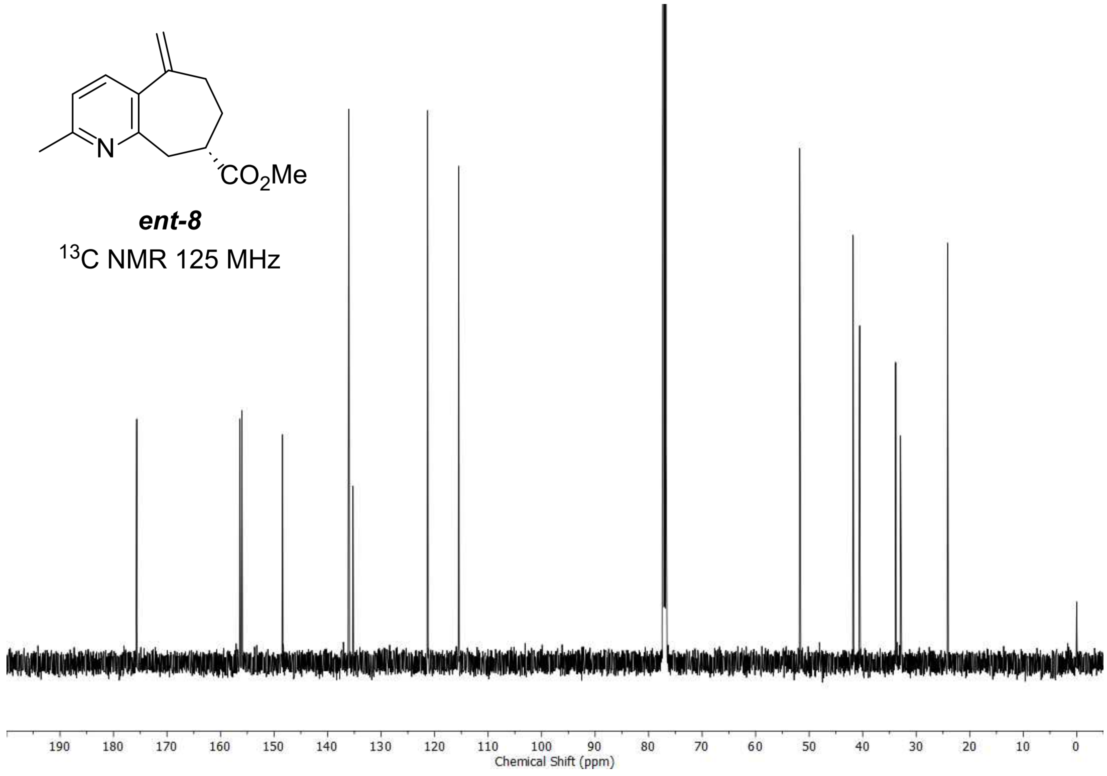

Figure S24. ^{13}C NMR Spectrum of ***ent-8***

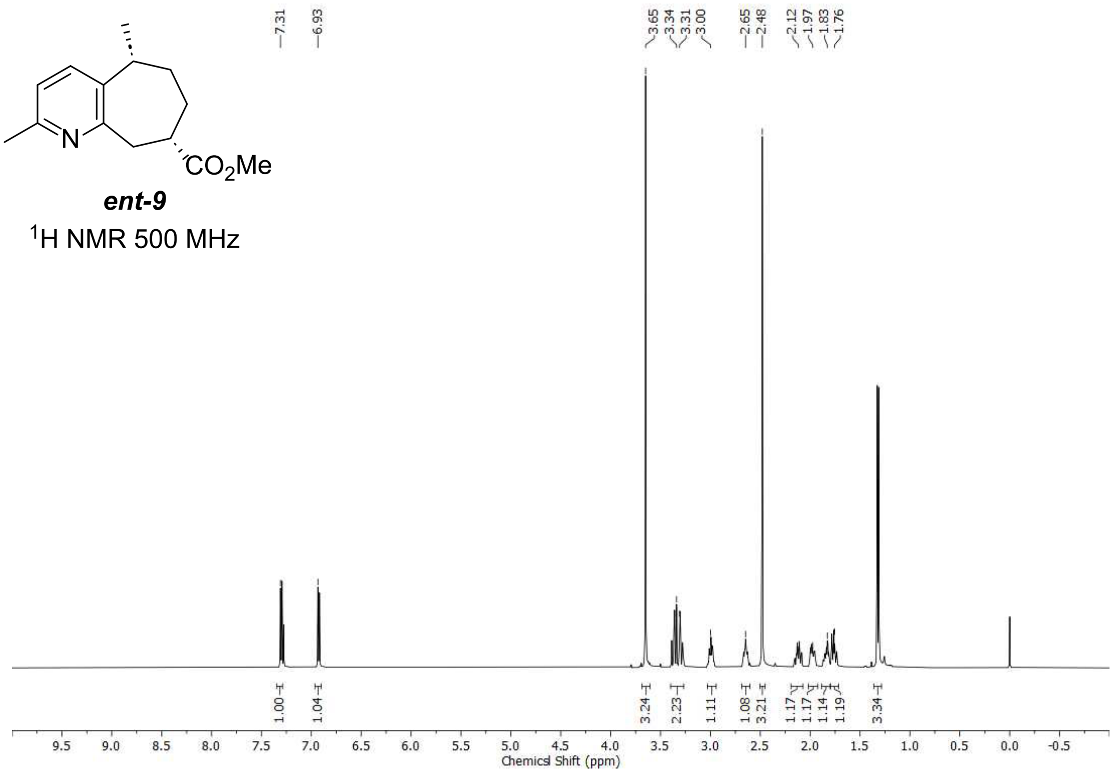

Figure S25. ^{1}H NMR Spectrum of ***ent-9***

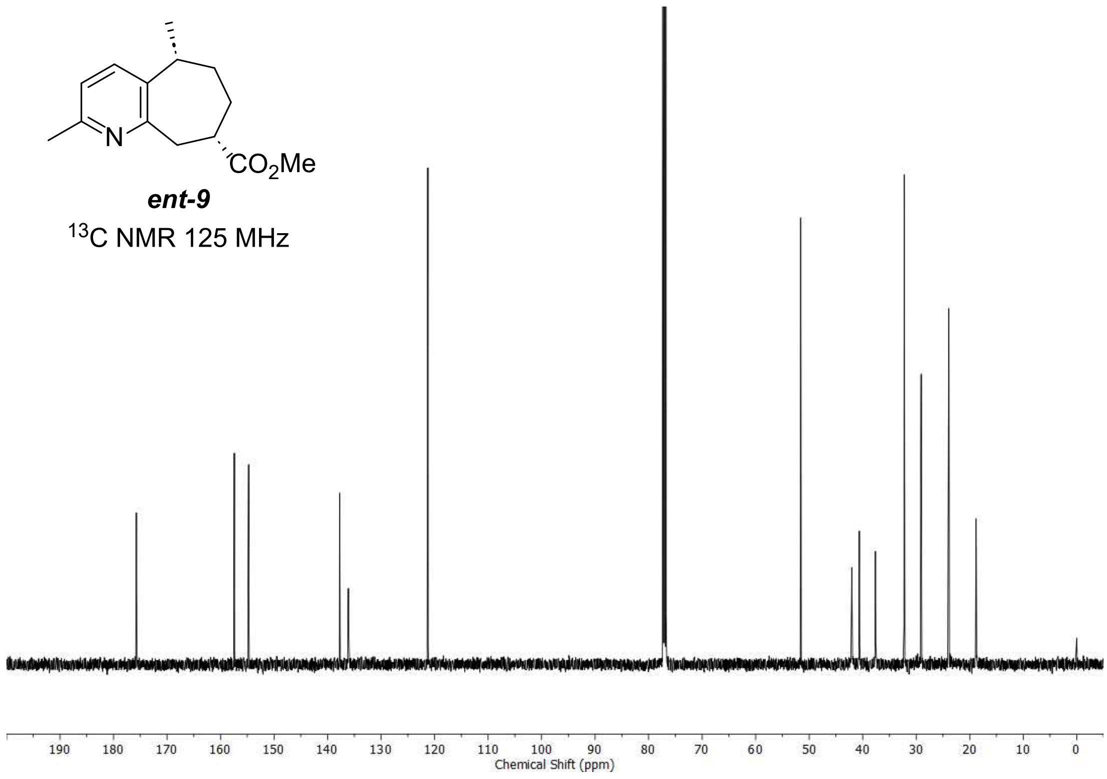

Figure S26. ^{13}C NMR Spectrum of ***ent-9***

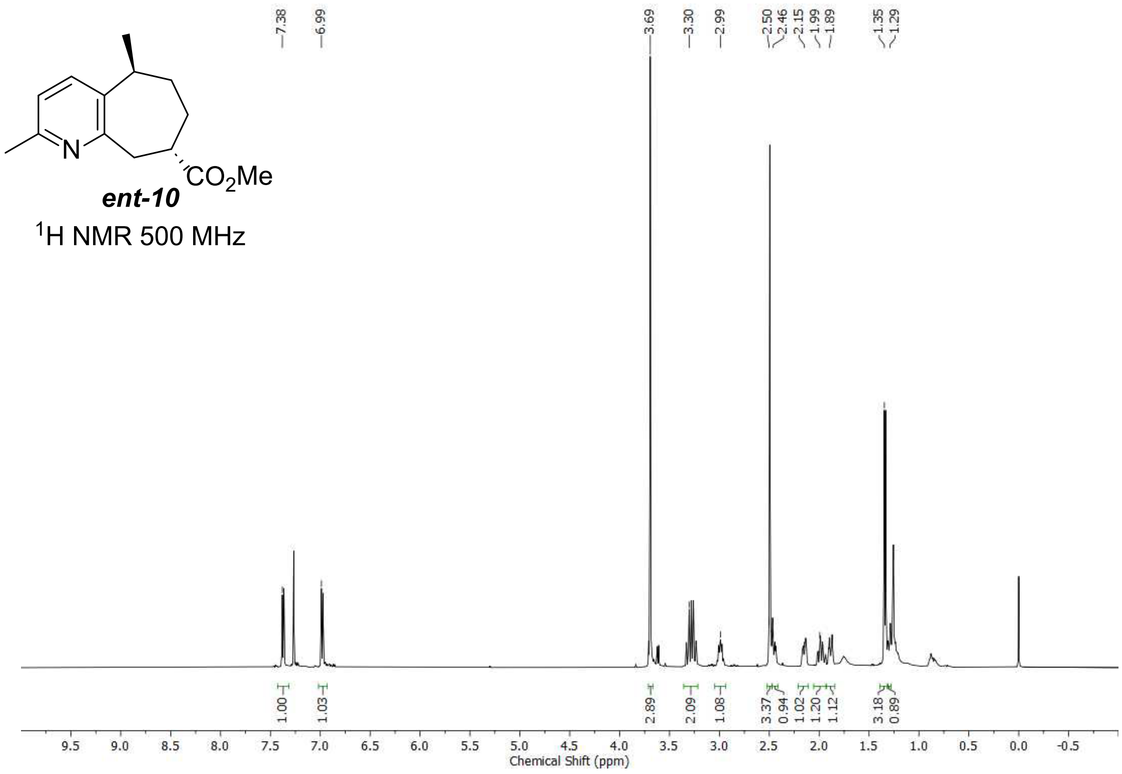

Figure S27. ^{1}H NMR Spectrum of ***ent-10***

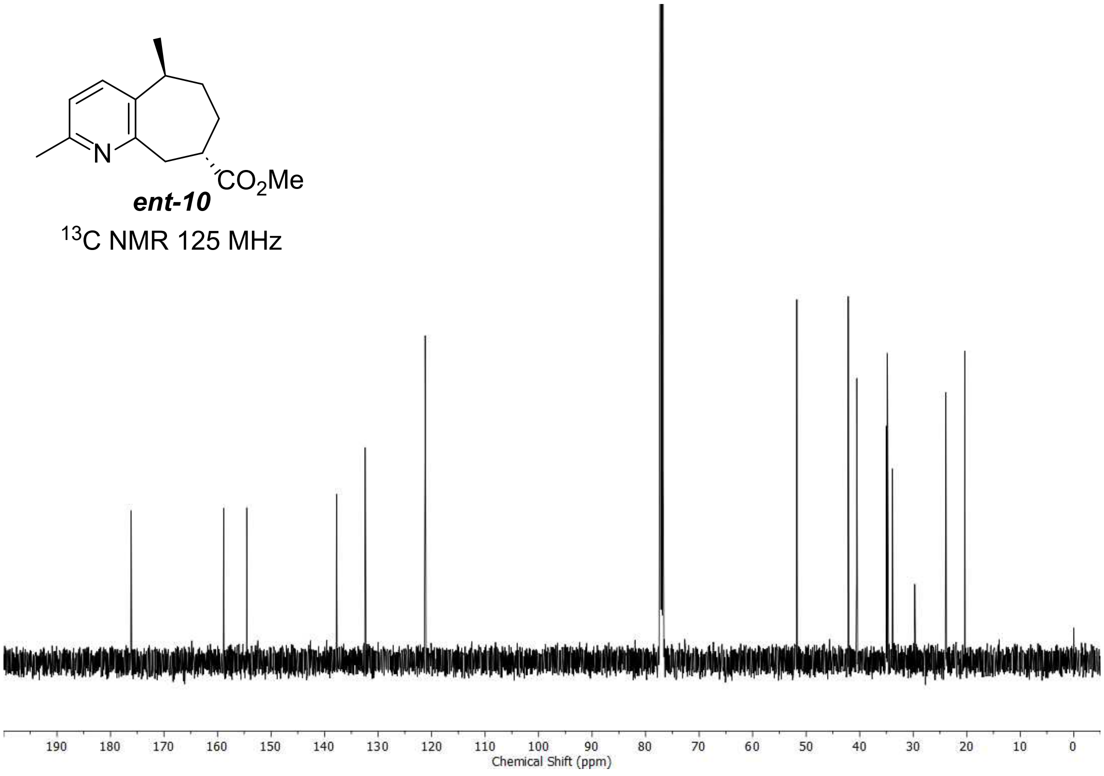

Figure S28. ^{13}C NMR Spectrum of ***ent-10***

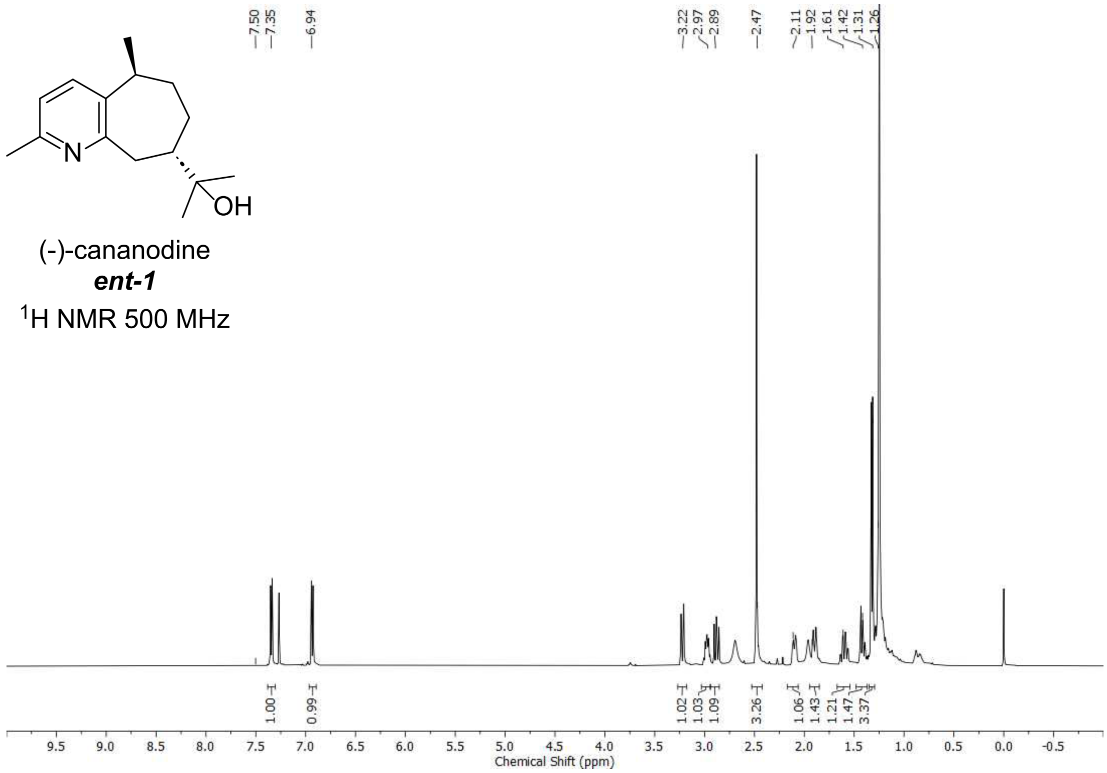

Figure S29. ^{1}H NMR Spectrum of (-)-Cananodine (***ent-1***)

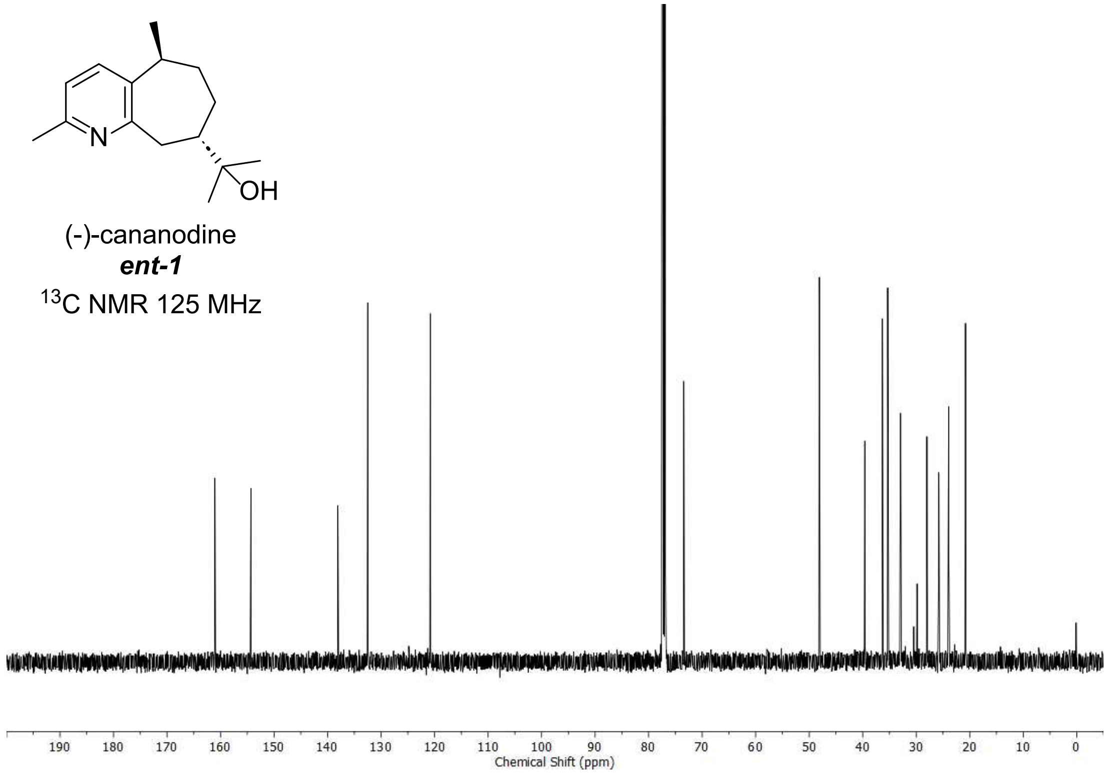

Figure S30. ^{13}C NMR Spectrum of (-)-Cananodine (***ent-1***)
